# The influence of board, CEO, and audit committee chairman busyness on the value relevance of accounting information in Saudi listed firms

**DOI:** 10.1371/journal.pone.0315886

**Published:** 2025-01-15

**Authors:** Abdulrahman Alomair, Abdulaziz S. Al Naim

**Affiliations:** Accounting Department, Business School, King Faisal University, Al-Ahsa, Saudi Arabia; KAIST: Korea Advanced Institute of Science and Technology, REPUBLIC OF KOREA

## Abstract

This study investigates the impact of board, CEO, and audit committee chairman busyness on the value relevance of accounting information in Saudi listed firms from 2018 to 2022. Utilizing a data of 125 Saudi firms, the research investigates how the busyness of these key governance figures influences the relevance of earnings per share (EPS) and book value per share (BVPS). The findings reveal that board and audit committee chairman busyness significantly enhance the value relevance of EPS, suggesting that their broader networks and strategic oversight improve financial reporting. Conversely, CEO busyness negatively affects the value relevance of BVPS, indicating that divided attention and reduced managerial oversight hinder performance monitoring. These results underscore the dual roles of governance figures and their influence on financial reporting quality. These results highlight the dual effects of governance busyness on financial reporting quality. The study provides novel insights into an emerging market context, offering practical recommendations for policymakers and corporate leaders in line with Saudi Arabia’s Vision 2030. It emphasizes the need for regulatory frameworks to manage director workloads, ensuring enhanced financial reporting and governance effectiveness.

## 1. Introduction

Understanding the value relevance of accounting information is crucial for evaluating the usefulness of financial reporting in firm valuation. As financial markets become increasingly complex and interconnected, the demand for high-quality accounting information that accurately reflects a firm’s economic reality has grown [1]. This topic has gained substantial interest among researchers, particularly in the context of corporate governance and the role of board, CEO, and audit committee chairman busyness [2]. Effective corporate governance mechanisms are essential to increase transparency, accountability, and the integrity of financial reporting [3]. However, the busyness of key governance figures has emerged as a critical issue, with significant implications for their ability to provide effective oversight [4].

The busyness of directors and executives can yield both beneficial and adverse impacts on governance practices. Busy directors bring extensive networks, diverse perspectives, and valuable experience that can support strategic oversight and decision making [5]. However, excessive commitments may lead to divided attention and reduced effectiveness in monitoring management, potentially compromising the quality of financial reports [6]. These dynamics underscore the importance of examining how the busyness of board members, CEOs, and audit committee chairmen impacts the value relevance of accounting information, particularly in emerging markets like Saudi Arabia.

This study makes several key contributions to the literature. First, it provides empirical evidence from Saudi Arabia, an emerging market with unique governance challenges, thereby enriching the global understanding of corporate governance’s role in value relevance [7, 8]. Second, this research extends the current literature on governance busyness by specifically examining the effects of board, CEO, and audit committee chairman busyness on the value relevance of accounting information, a topic that has been underexplored in the Middle Eastern context [9]. Third, by incorporating the institutional changes brought about by Saudi Arabia’s Vision 2030, the study adds a timely perspective on how regulatory reforms shape corporate behavior and financial reporting practices [10]. Lastly, the study offers practical recommendations for decision makers, corporate leaders, and investors, emphasizing the need for balanced governance structures to enhance financial reporting quality [11].

Saudi Arabia presents an intriguing case for this study due to its significant role as the leading country in the Middle East and the largest producer of oil globally [12]. As the largest Islamic country that has never been colonized by European powers, Saudi Arabia offers a unique perspective on the implementation of corporate governance practices in a non-Western context [13]. The reliance on income from the oil and gas industry has driven the Saudi government to launch Vision 2030, which aims to diversify its economic resources and reduce dependency on oil. These reforms have improved Saudi Arabia’s ease of doing business ranking, placing it second in the GCC region after Bahrain in 2020, and significantly strengthened the protection of minority investors, ranking third worldwide [14]. These factors make Saudi Arabia an ideal setting to examine how board, CEO, and audit committee chairman busyness influences financial reporting and firm valuation.

The primary objective of this study is to investigate the influence of board, CEO, and audit committee chairman busyness on the value relevance of accounting information in Saudi listed firms. Specifically, the study aims to assess whether busy boards positively influence the value relevance of accounting information (H1), busy CEOs negatively influence the value relevance of accounting information (H2), and busy audit committee chairmen positively influence the value relevance of accounting information (H3).

The analysis of this study depends on 125 non-financial firms listed on the Saudi Exchange from 2018 to 2022. Our findings reveal that board and audit committee chairman busyness positively impact the value relevance of earnings per share (EPS) by enhancing strategic oversight and leveraging extensive networks, while CEO busyness negatively affects the value relevance of book value per share (BVPS) due to divided attention and reduced effectiveness in monitoring management [6, 15].

This research offers significant contributions to existing literature, notably by supplying empirical data from Saudi Arabia, an emerging market characterized by distinctive governance challenges, thereby enriching the global understanding of corporate governance’s role in value relevance [7, 8]. Second, this study expands on the existing literature concerning governance busyness by specifically examining the effects of board, CEO, and audit committee chairman busyness on the value relevance of accounting information, a topic that has been underexplored in the Middle Eastern context [9]. Third, by incorporating the institutional changes brought about by Saudi Arabia’s Vision 2030, the study adds a timely perspective on how regulatory reforms shape corporate behavior and financial reporting practices [10]. Lastly, the study offers practical recommendations for policymakers, corporate leaders, and investors, emphasizing the need for balanced governance structures to enhance financial reporting quality [11].

The organization of this paper is as follows: Section 2 delves into the institutional framework of Saudi Arabia, emphasizing the corporate governance environment and the importance of Vision 2030. Section 3 presents the literature review and this study’s hypotheses. Section 4 outlines the methodology. While the findings of this study in Section 5. Section 6 shows the interpretation of the findings, and Section 7 concludes the study.

## 2. The Saudi Arabian context

Saudi Arabia’s corporate governance landscape has undergone substantial transformations in recent years, particularly following the introduction of the New Company Regulations and Corporate Governance Regulations (CGR) in 2017. These reforms are part of a broader effort to align Saudi corporate governance with international best practices, enhancing transparency, accountability, and investor confidence, which are crucial for achieving the goals set forth in Vision 2030 [10].

Article 83.1 of the New Regulations enforces board independence by mandating the creation of essential board committees and preventing the chair from holding executive roles. Despite this, the regulation does not impose limitations on holding multiple directorships, which can lead to concerns regarding board busyness. The intense demand for skilled directors, further intensified by strict criteria for expertise in fields like accounting and finance, contributes to a shortage of eligible candidates [13]. Consequently, this shortage has led to a dependence on a limited pool of experienced professionals serving on various boards [16]. In Saudi Arabia, the common family ownership models significantly add to the issue of board busyness, with family members frequently occupying multiple board positions within related firms. By the close of 2017, data shows that 83% of the 43 publicly listed family-controlled firms had a family member as chairman, and family members made up 32% of all board seats [17]. These family-owned corporations are a crucial part of the Saudi economy, contributing over $216 billion to the GDP [18].

The CGR introduced by the Capital Market Authority (CMA) emphasizes the importance of board independence, requiring at least two independent directors and stipulating that at least one-third of the board should comprise independent members [17]. For instance, STC adjusted its board composition to comply with these new guidelines in 2017 [19]. Additionally, the CGR mandates that boards hold at least two annual meetings to ensure effective governance, leading firms like Kingdom Holding to increase their board meetings from one in 2016 to two in 2017 [20]. Even with these reforms in place, the problem of board busyness persists, driven by a restricted pool of qualified directors and a cultural focus on prestige that leads to prominent directors being appointed to multiple boards [21]. Additionally, directors frequently have lengthy tenures and multiple board roles, exacerbating their [8]. As part of Vision 2030’s objective to transition Saudi Arabia from an oil-dependent economy to a more diversified and investment-attractive market, the impact of busyness among board members, CEOs, and audit committee chairpersons on the value relevance of accounting information is increasingly critical. Vision 2030 seeks to increase confidence in the Saudi stock market to attract foreign direct investment (FDI) and foster a transparent business environment [10]. The busyness of these key governance figures can significantly impact the reliability and usefulness of financial reports, which consequently affects investor trust and firm valuation.

Busy board members, CEOs, and audit committee chairmen may struggle to devote adequate time and attention to their duties, potentially compromising the quality of oversight and decision-making. This can lead to less reliable financial reporting, affecting investors’ ability to make informed investment decisions. Reliable and transparent financial reporting is essential for building investor confidence, which is a key component of Vision 2030’s strategy to attract FDI and diversify the economy. Moreover, the effectiveness of governance mechanisms in ensuring high-quality financial reporting is critical for firm valuation. Investors rely on accurate and timely financial information to assess firm performance and make investment decisions. If board, CEO, and audit committee chairman busyness adversely affects the quality of financial reports, it could undermine investor confidence and negatively impact firm valuation.

The institutional context of Saudi Arabia, characterized by significant corporate governance reforms and unique ownership structures, provides a relevant setting for examining the influence of board, CEO, and audit committee chairman busyness on the value relevance of accounting information. Grasping the influence of these elements on corporate governance and financial reporting is essential for boosting the effectiveness and dependability of boards, thus aiding the wider objectives of Vision 2030.

## 3. Literature review and hypothesis development

### 3.1. Corporate governance and value relevance of accounting information

The concept of value relevance in accounting information pertains to how the figures in financial statements encapsulate data that affects equity values, which in turn assists investors in making knowledgeable investment decisions [22]. This concept is crucial for firm valuation as it reflects the extent to which financial metrics such as earnings and book value influence market indicators like share prices and returns. Reliable and transparent accounting information is essential for assessing a firm’s overall value, as it provides insights into the firm’s performance, risk management, and operational efficiency [1].

Good corporate governance practices play a vital role in enhancing the quality of accounting information. Effective governance mechanisms are essential to ensure that management actions reflect the interests of shareholders and other stakeholders. These practices help reduce information asymmetry, mitigate agency costs, and improve the credibility of financial reporting [23]. High-quality financial reporting, in turn, enhances investor confidence and can lead to better firm valuation. Further, Big Data (BD) integration into financial accounting enhances reporting quality, risk management, and decision-making, ultimately improving firm sustainability and the value relevance of accounting information [24].

The connection between corporate governance and firm valuation is extensively recognized in academic research. Practices such as maintaining board independence, managing appropriate board sizes, and conducting regular board meetings are linked to enhance financial reporting quality and transparency [3]. This enhancement in transparency and accountability can positively impact the usefulness of financial numbers, as investors are more inclined to trust and depend on financial statements that are deemed accurate and impartial [25]. Independent directors, for instance, are noted for their effectiveness in overseeing management, curbing earnings manipulation, and ensuring financial statements accurately represent the firm’s true economic state [26].

Agency Theory posits that the busyness of governance figures like CEOs can lead to higher agency costs due to their split focus and insufficient oversight, which consequently could reduce the quality of financial reports [23]. According to this theory, there is an anticipated negative correlation between CEO busyness and the value relevance of accounting information, particularly in terms of book value per share (BVPS), because busy CEOs might not monitor balance sheet items effectively [27].

Conversely, Resource Dependence Theory [5] offers an alternative perspective, positing that busier board members and audit committee chairmen can leverage their experience, extensive networks, and external resources to support firm decision-making and financial oversight. This theory predicts a positive relationship among the busyness of these governance figures and the value relevance of earnings per share (EPS), as they bring valuable insights from their external roles that improve financial reporting quality [11]. By integrating these two theoretical perspectives, this research offers a detailed analysis of how various forms of governance busyness influence the significance of financial information in Saudi Arabia’s emerging market [8, 28].

While both agency theory and resource dependence theory provide valuable insights into the role of governance in financial reporting, the applicability of each theory is context dependent. Agency theory is more likely to dominate in situations where governance structures are weak, or where directors and executives face significant time constraints due to multiple commitments. In such cases, the negative effects of busyness—such as reduced oversight and increased agency costs—are more pronounced. For example, directors who are overcommitted may find themselves unable to adequately oversee management, which can result in poorer financial reporting quality and decreased the usefulness of financial numbers [15, 27]. This issue is especially pertinent in firms with concentrated family ownership, where governance structures are often less robust, heightening the dangers of insufficient supervision [9]. However, resource dependence theory is more applicable in contexts where governance structures are strong, and the directors and executives have access to support systems that mitigate the risks of over-commitment. Under such conditions, busy directors can leverage their experience, extensive networks, and external resources to support strategic decision making and financial oversight [5, 11]. Their ability to bring in best practices from other boards and industries can support the financial reports quality, thereby increasing the usefulness of financial numbers. This theory is particularly relevant in firms with well-established governance practices, such as independent boards and audit committees, where the benefits of busyness can outweigh the risks [11].

Thus, the balance between agency theory and resource dependence theory depends on the governance environment of the firm. In firms with strong governance mechanisms, resource dependence theory’s predictions of positive outcomes from busyness are more likely to hold. Conversely, in firms with weak governance, agency theory’s predictions of negative outcomes from busyness are more applicable. This dual framework allows us to build different types of hypotheses to gain a better understanding of this study.

Empirical evidence from international studies supports these theoretical arguments. For instance, studies by [26, 29] demonstrate that board independence is associated with reduced earnings manipulation, thereby improving financial reporting quality and increasing the value relevance of accounting information. Similarly, [30] found that larger boards help to restrain manipulative practices, and [31] reported a positive moderating effect of board size on the earnings-return connection for UK firms in terms of value relevance. Additionally, [32] found that more active boards increase intellectual capital disclosures in Taiwanese firms.

Studies from Arab and GCC nations also highlight the crucial role of corporate governance practices. For instance, research by [33] across GCC countries, including Saudi Arabia, shows a positive correlation between audit quality—determined by the engagement of Big Four auditors—and corporate governance structures, demonstrating a mutually beneficial relationship between the two. Additionally, a study by [9] in Saudi Arabia indicates that board independence correlates with increased real earnings management, suggesting that independent directors play a significant role in overseeing financial reports and enhancing the usefulness of financial numbers.

Specifically in Saudi Arabia, the importance of corporate governance practices is highlighted by several studies. [34] demonstrated that board independence increases the value relevance of accounting metrics such as earnings and book values. [21] found that effective corporate governance practices, including board independence and audit committee diligence, improve financial reporting quality and investor confidence. These findings underscore the critical role of corporate governance in enhancing the reliability and usefulness of financial information, which is essential for attracting foreign direct investment (FDI) and supporting the broader goals of Vision 2030.

Despite the extensive research on corporate governance, there is a notable lack of studies examining the specific impact of board, CEO, and audit committee chairman busyness on this relationship, both globally and within the Saudi context [8, 35]. The literature reveals a notable gap concerning the impact of the busyness of key governance figures like directors, CEOs, and audit committee chairmen, which significantly influences their ability to oversee financial reporting and ensure the integrity of disclosures. Studies such as [4, 15] highlight that these busy governance figures might find it challenging to allocate sufficient time and attention to their roles, which could impair the quality of oversight and decision-making. Conversely, research by [6] and others suggest that such individuals might use their broad networks and insights from holding multiple roles to improve governance practices and the quality of financial reporting [36].

Given the ambitious goals of Vision 2030 to transform Saudi Arabia into a diversified and investment-friendly market, understanding the influence of board, CEO, and audit committee chairman busyness on the value relevance of accounting information is crucial. Reliable and transparent financial reporting is essential for building investor confidence and attracting FDI, which are key components of Vision 2030’s strategy to move away from an oil-based economy. Consequently, this research seeks to address this void by exploring how the busyness of these key governance figures impacts the reliability and usefulness of financial reporting, ultimately affecting firm valuation and investor trust in Saudi listed firms. This study will offer important perspectives for maker decisions and corporate leaders seeking to enhance governance practices and support the broader economic goals of Vision 2030.

### 3.2. Busy board and firm value

The concept of board busyness, generally described by directors holding three or more board positions, raises important concerns about its effects on the quality and value relevance of accounting information [37–39]. Given the importance of accounting quality for equity investors—who rely on this data to make informed decisions and accurately assess firm value—the impact of a busy board on these factors is a critical issue to explore.

While a busy board can influence the quality of accounting information in various ways, there are potential benefits. Directors who hold multiple board positions may possess a wealth of knowledge, experience, and broad networks, enhancing their ability to advise and strategically oversee the firm [40]. Their varied perspectives might lead to more thorough evaluations and balanced decision-making, potentially elevating the quality of financial reporting. This enhancement in reporting quality could positively impact the value relevance of accounting information, as transparent and dependable financial statements are crucial for investors making informed decisions [1].

Nonetheless, time constraints significantly hinder the capacity of busy directors to adequately manage their advisory and oversight responsibilities. Directors with multiple commitments may struggle to devote adequate time and attention to their responsibilities on each board, potentially compromising the quality of oversight and decision-making [6]. This lack of engagement can lead to less effective monitoring of management, increased earnings manipulation, and ultimately, lower quality financial reporting [15]. Consequently, the perceived reliability of accounting information may diminish, leading to suboptimal investment decisions and negatively impacting firm valuation.

The influence of a busy board on a company’s value can be analyzed using two key theoretical frameworks (agency theory and resource dependence theory). The applicability of these two theories depends on the contextual factors within the firm. Agency theory is more likely to apply when directors are over-committed and the governance structures are weak or insufficient to provide the necessary support. In such situations, the negative consequences of busyness—such as reduced monitoring and higher agency costs—are more likely to dominate [6, 27]. This is especially true in family-owned firms, where board independence may be limited, and the oversight of management can be compromised [9].

Furthermore, resource dependence theory posits that when governance structures are robust—characterized by independent boards, well-functioning audit committees, and regular board meetings—busy directors can enhance the strategic oversight of the firm. In these environments, directors can draw on their external networks and experiences to provide valuable resources and improve decision-making, which consequently improves the quality of financial reporting [11]. Therefore, the extent to which board busyness positively or negatively impacts value relevance depends on the firm’s governance quality. National culture influences sustainability reporting, with busy boards moderating this relationship. Firms in high power-distance cultures disclose more, indicating that board busyness and cultural context jointly affect governance and reporting [41].

The evidence regarding the impact of busy boards on firm performance reveals conflicting findings. [42] analyzed U.S. firms from 1999 to 2011, concluding that firms with busy boards might see improved performance due to directors’ extensive experience and connections. Similarly, [43] found that busy boards could boost firm performance through their broad networks. Conversely, [27], who studied U.S. firms from 1998 to 2002, argued that busy boards might lead to weaker monitoring, resulting in lower quality financial reporting and reduced firm performance. Additionally, [44] discovered that busy directors in U.S. firms correlated with negative abnormal returns for acquiring firms, indicating that over-committed directors might not always act in the best interests of shareholders.

In Arab and GCC countries, studies have shown varied impacts of board busyness. [33] identified a positive correlation between audit quality and corporate governance structures in GCC countries, indicating that busy directors may enhance the quality of financial reporting. Similarly, in a study of firms in Jordan, [45] found that busy boards reduced audit quality, highlighting the potential detrimental impacts of board busyness on financial oversight. [46] conducted a study in Iran that revealed a negative correlation between board busyness and firm productivity, particularly significant in firms requiring intensive monitoring.

Specifically, in the Saudi Arabian context, the effect of board busyness on the value relevance of accounting information is especially relevant due to the prevalent family ownership and the changing landscape of corporate governance. [9] discovered that in Saudi firms, busy directors are linked to increased real earnings management, indicating busy directors may not effectively monitor financial reporting, leading to lower quality disclosures. [34] demonstrated that strong corporate governance practices, including board independence and diligent audit committee activities, enhance the value relevance of accounting metrics such as earnings and book values. Additionally, [28] found no significant link between various attributes of the audit committee and audit quality in Saudi firms, implying that mere compliance does not necessarily ensure effective governance.

The unique governance context in Saudi Arabia suggests that the impact of busy boards on the value relevance of accounting information could vary. While some studies indicate that busy boards might struggle to provide effective oversight, others argue that the busy directors with high expertise and networking could boost governance practices and improve the quality of financial disclosures. Given the ambitious goals of Vision 2030 to transform Saudi Arabia into a diversified and investment-friendly market, there is a strong case to be made that the benefits of the expertise and networks of busy directors could outweigh the challenges posed by their multiple commitments. This could ultimately enhance the usefulness of financial numbers.

Therefore, this study hypothesizes that:

*H1*: *Busy boards are likely to positively influence the value relevance of accounting information in Saudi listed firms*.

### 3.3. Busy CEOs and firm value

CEO busyness refers to the situation where Chief Executive Officers hold multiple board positions concurrently. This phenomenon raises concerns about their ability to effectively manage their primary responsibilities. A CEO is typically considered "busy" if they hold two or more directorships in addition to their CEO role [47, 48]. The implications of CEO busyness on firm value and the quality of accounting information are critical for understanding corporate governance dynamics and investor decision-making.

The quality of accounting information is paramount for equity investors as it underpins informed investment decisions and accurate firm valuations. However, the dual commitments of busy CEOs can significantly impact the quality of this information. While busy CEOs might bring extensive networks and valuable insights to their firms, their limited time and attention can impede effective oversight of financial reporting processes [6]. This divided focus can lead to increased earnings manipulation and lower financial reporting quality, thereby diminishing the value relevance of accounting information [15].

Agency theory provides a framework for understanding the potential negative impacts of CEO busyness. According to this theory, busy CEOs may incur higher agency costs due to their inability to effectively monitor and control managerial behavior [23]. This lack of oversight can result in opportunistic behaviors by managers, increasing the likelihood of financial misreporting. Resource dependence theory, while acknowledging the potential benefits of a CEO’s external networks, suggests that the over-commitment of a busy CEO can dilute their effectiveness in contributing to their primary firm [5].

The negative effects of CEO busyness predicted by agency theory are particularly relevant in firms where the CEO plays a dominant role in both strategic decision-making and operational oversight, and where there are fewer checks and balances in place. In such firms, the CEO’s divided attention can lead to less effective monitoring of financial reporting and higher risks of earnings manipulation, resulting in lower value relevance [27]. This is likely to occur in firms with weaker governance structures or high CEO dominance, where oversight by other executives or the board is limited.

Conversely, resource dependence theory is more applicable in firms where the CEO has access to strong internal governance mechanisms and can delegate operational responsibilities effectively. In such cases, the CEO’s external networks and insights from serving on multiple boards can add value to the firm by improving strategic oversight and enhancing the quality of financial disclosures [11]. Therefore, the impact of CEO busyness on value relevance is contingent on the strength of the firm’s governance framework and the CEO’s ability to manage their dual commitments effectively.

Empirical evidence on the impact of busy CEOs largely supports these theoretical concerns. [27] found that U.S. firms with busy CEOs exhibited weaker governance structures, which led to lower profitability and market-to-book ratios during 1998–2002. Similarly, [49] reported that U.S. banks with busy directors and CEOs faced higher risks. [44] also found that busy directors, including CEOs, in U.S. firms were associated with negative abnormal returns for acquiring firms, suggesting that over-committed directors may not act in the best interests of shareholders.

In the context of Arab and GCC countries, studies have also indicated the potential drawbacks of CEO busyness. [45] found that CEO duality in Jordanian firms, where the CEO also serves as board chair, reduced audit quality, highlighting the negative effects of CEO busyness on financial oversight. [46] in Iran reported a negative association between CEO busyness and firm productivity, which is especially significant for firms requiring high levels of monitoring.

Specifically, in the Saudi Arabian context, the unique corporate governance landscape, characterized by concentrated family ownership, amplifies the challenges posed by CEO busyness. [9] found that busy directors in Saudi firms are associated with greater real earnings management, indicating that busy CEOs may similarly fail to effectively monitor financial reporting, leading to lower quality disclosures. [34] demonstrated that robust corporate governance practices can enhance the value relevance of accounting metrics such as earnings and book values. However, [28] found no significant link between CEO busyness and audit quality in Saudi firms, suggesting that simply adhering to compliance standards does not ensure effective governance.

Given the ambitious goals of Vision 2030 to transform Saudi Arabia into a diversified and investment-friendly market, the negative implications of CEO busyness on the quality of financial disclosures could undermine investor confidence and hinder firm valuation. The prevailing evidence suggests that the divided attention and reduced oversight capabilities of busy CEOs are more likely to lead to lower quality financial reporting, which is detrimental in the context of Saudi Arabia’s strategic economic objectives. During crises like COVID-19, corporate sustainable development (CSD) strengthens financial performance, highlighting the importance of effective governance in supporting value relevance during challenging times [50].

Therefore, this study hypothesizes that:

*H2: Busy CEOs are likely to negatively influence the value relevance of accounting information in Saudi listed firms.*

### 3.4. Busy audit committee chairman and firm value

The audit committee chairman plays a critical role in overseeing a company’s financial reporting and ensuring the integrity of its accounting processes. A "busy" audit committee chairman, who holds multiple board positions, often raises concerns about their capacity to effectively fulfill their responsibilities. However, there are significant arguments and empirical evidence suggesting that a busy audit committee chairman can positively influence the usefulness of financial numbers.

Resource dependence theory posits that busy audit committee chairmen can leverage their extensive networks and diverse experiences to bring valuable insights and best practices to the firms they serve. Their broad exposure to different industries and governance practices can enhance their ability to effectively oversee financial reporting and internal controls [11]. This can lead to more rigorous financial oversight, ensuring high-quality disclosures that are critical for investors making informed decisions [5].

Empirical studies support this view. For instance, [51] found that multiple directorships positively influence the level of disclosure among Malaysian firms, indicating that busy directors can enhance transparency and reporting quality. Similarly, [52] showed that the extent of disclosures is enhanced when multiple directorships exist. These findings suggest that the experience and knowledge gained from serving on multiple boards can improve the quality of financial information provided to investors.

In the context of Saudi Arabia, where corporate governance practices are evolving and the market is striving to attract more foreign investment, the presence of experienced and well-connected audit committee chairmen can be particularly beneficial. The unique challenges posed by concentrated family ownership and the need for robust oversight mechanisms make the role of the audit committee chairman even more critical. Studies such as those by [9, 33] highlight the importance of effective audit committee practices in enhancing financial reporting quality in Saudi firms.

The reputation and standing of busy audit committee chairmen can instill greater confidence among investors. Their participation is considered as a positive signal of the firm’s commitment to high levels of governance and transparency, which may enhance the firm’s credibility and attractiveness to investors [53]. This is particularly relevant in the context of Saudi Arabia’s Vision 2030, which aims to diversify the economy and boost investor confidence through improved governance practices.

While some studies raise concerns about the potential drawbacks of busyness due to time constraints [27, 54], busy directors with high expertise and networking often outweigh these challenges. Their ability ensure rigorous financial oversight can lead to more reliable and transparent financial disclosures, ultimately benefiting investors and enhancing firm valuation. Voluntary risk disclosures are enhanced by larger audit committees, improving firm value through better governance and transparency [55].

Therefore, this study hypothesizes that:

*H3: Busy audit committee chairmen are likely to positively influence the value relevance of accounting information in Saudi listed firms.*

## 4. Research methodology

### 4.1. Sample selection and data collection

This sample of this study covers the period between 2018 and 2022. This period was chosen to assess the impact of the new corporate governance regulations introduced in 2017, which required detailed disclosure of directors’ characteristics [56]. A balanced panel date (625 firm-year observations) was employed to control for specific firm characteristics, thus improving comparability and mitigating endogeneity issues [57]. Financial firms were excluded from the sample, due to their unique governance requirements, and firms with non-standard fiscal years were omitted to ensure consistency and comparability [58]. Data concerning governance were obtained from the annual reports manually, sourced from the Saudi Exchange website (www.saudiexchange.sa). Financial information was obtained from Bloomberg. This methodology provides a solid foundation for analyzing how the busyness of board members, CEOs, and audit committee chairmen impacts the value relevance of accounting data in Saudi firms.

### 4.2. Model specification

In this research, the Generalized Linear Model (GLM) is utilized to analyze the effect of busyness of board members, CEOs, and audit committee chairmen on the value relevance of accounting data in Saudi listed firms. The selection of the GLM method is due to its efficacy in managing residual patterns, variance fluctuations, and non-stationarity, which ensures more accurate results [59]. This methodological choice contrasts with earlier studies e.g., [2, 60–62] that applied Ordinary Least Squares (OLS) regression. OLS may lead to biased and inefficient outcomes if there are violations in assumptions like residual normality and homoscedasticity.

To test the study’s hypotheses, three extended price models based on [22] are estimated. These models capture the relationship between the busyness of board members, CEOs, and audit committee chairmen, and the value relevance of accounting information. Consistent with prior studies [63], the number of shares outstanding is used as a deflator to mitigate the issue of scale effect. The panel data structure allows for modeling behavioral differences across firms and time periods [64].

#### 4.2.1. Model 1: Impact of board busyness (H1)

To test the first hypothesis (H1) regarding the impact of busy board members on the value relevance of key accounting information, we use the following extended price model:

$$P_{it}=a_{0}+b_{1}{BVPS}_{it}+b_{2}{EPS}_{it}+b_{3}{BUSYBOD}_{it}+b_{4}{BVPS}_{it}*{BUSYBOD}_{it}+b_{5}{EPS}_{it}*{BUSYBOD}_{it}+b_{6}{BIND}_{it}+b_{7}{BSIZE}_{it}+b_{8}{BMEET}_{it}+b_{9}{FSIZE}_{it}+b_{10}\;{FLEV}_{it}+b_{11}\;{ROA}_{it}+b_{12}\;{MVTBV}_{it}+b_{13}{FAGE}_{it}{+b_{14}{LOSS}_{it}+b_{15}{BIG4}_{it}+Industry\;dummies+Year\;dummies+e}_{it}$$ (1)

The main focus is on coefficients *b*_4_ and *b*_5_, which show the interaction effect of board busyness on the key accounting information of book value of equity and earnings.

#### 4.2.2. Model 2: Impact of CEO busyness (H2)

To test the second hypothesis (H2) regarding the impact of busy CEOs on the value relevance of key accounting information, the following model is specified:

$$P_{it}=a_{0}+b_{1}{BVPS}_{it}+b_{2}{EPS}_{it}+b_{3}{BUSYCEO}_{it}+b_{4}{BVPS}_{it}*{BUSYCEO}_{it}+b_{5}{EPS}_{it}*{BUSYCEO}_{it}+b_{6}{BIND}_{it}+b_{7}{BSIZE}_{it}+b_{8}{BMEET}_{it}+b_{9}{FSIZE}_{it}+b_{10}\;{FLEV}_{it}+b_{11}\;{ROA}_{it}+b_{12}\;{MVTBV}_{it}+b_{13}{FAGE}_{it}{+b_{14}{LOSS}_{it}+b_{15}{BIG4}_{it}+Industry\;dummies+Year\;dummies+e}_{it}$$ (2)

The interaction coefficients *b*_4_ and *b*_5_ are of key interest as they show the impact of CEO busyness on the value relevance of book value of equity and earnings.

#### 4.2.3. Model 3: Impact of audit committee chairman busyness (H3)

To test the third hypothesis (H3) regarding the impact of audit committee chairman busyness, the following model is employed:

$$P_{it}=a_{0}+b_{1}{BVPS}_{it}+b_{2}{EPS}_{it}+b_{3}{ACCBUSY}_{it}+b_{4}{BVPS}_{it}*{ACCBUSY}_{it}+b_{5}{EPS}_{it}*{ACCBUSY}_{it}+b_{6}{ACIND}_{it}+b_{7}{ACSIZE}_{it}+b_{8}{ACMEET}_{it}+b_{9}{ACEXP}_{it}+b_{10}{FSIZE}_{it}+b_{11}\;{FLEV}_{it}+b_{12}\;{ROA}_{it}+b_{13}\;{MVTBV}_{it}+b_{14}{FAGE}_{it}{+b_{15}{LOSS}_{it}+b_{16}{BIG4}_{it}+Industry\;dummies+Year\;dummies+e}_{it}$$ (3)

The interaction coefficients *b*_4_ and *b*_5_ are essential for understanding the moderating effect of audit committee chairman busyness on the value relevance of book value of equity and earnings.

All models include industry and year dummies to account for heterogeneity and time-varying impacts. This approach ensures robust insights into the impact of board, CEO, and audit committee chairman busyness on the value relevance of accounting information in Saudi firms.

### 4.3. Variables measurements

This study investigates the influence of board, CEO, and audit committee chairman busyness on the value relevance of accounting information in Saudi listed firms. Here are the definitions and measurements of all variables used in the study, as summarized in Table 1.

**Table 1:** Variables definitions and measurements.

Name of the Variable	Acronym	Measurement	References	data source
dependent variable				
Share price	*P*	Share price four months after the end of fiscal year	[22, 62, 64].	Thomson Reuters DataStream
Independent variables				
Book values of equity per share	*BVPS*	Reported book values of equity per share at the end of fiscal year	[22, 37, 62, 64].	Thomson Reuters DataStream
Earnings per share	*EPS*	Reported earnings per share at the end of fiscal year	[22, 64–66].	
Busy Board Members	*BODBUSY*	Dummy variable for busy board, which takes the value of 1 if it has a director who holds five or more board appointments, and 0 otherwise	[67, 68, 69].	Annual Reports
Busy CEOs	*CEOBUSY*	Dummy variable (0,1) by taking 1 for CEOs who hold two or more board appointments, and 0 for CEOs who hold less than two other board appointments.	[47, 48, 70].	
Busy Audit Committee Chairman	*ACCBUSY*	Dummy variable for a busy audit committee chairman, which takes the value of 1 if the chairman holds two or more appointments on other public company boards concurrently during the year, and 0 otherwise	[4, 72–74].	
Control variables				
Board Independence	*BIND*	A proportion of independent to total board members	[45, 75–78].	Annual Reports
Board Size	*BSIZE*	A total count of board members	[23, 76, 78, 79], and [80].	
Board Meetings	*BMEET*	The total count of board meetings held during the fiscal year.	[76, 80–82].	
Audit Committee Independence	*ACIND*	A proportion of independent members versus the total members on the audit committee.	[83–87].	
Audit Committee Size	*ACSIZE*	A total count of audit committee	[88, 89].	
Audit Committee Meetings	*ACMEET*	a total number of audit committee meetings within fiscal year	[90, 91].	
Audit Committee Financial Expertise	*ACEXP*	A ratio of audit committee members with financial expertise (professional accounting or finance certification or experience as an accountant or CFO) to total members of audit committee	[35, 88, 92–94]	
Firm’s Size	*FSIZE*	The natural logarithm of total assets	[2, 95, 96].	Thomson Reuters DataStream
Firm’s Financial Leverage	*FLEV*	Debt-to-assets ratio	[2, 45, 97].	
Firm Profitability	*ROA*	Net profit to total assets ratio	[98, 99].	
Valuation Ratios	*MVTBV*	Market-to-Book Value ratio	[97, 98, 100].	
Firm Age	*FAGE*	The length of time since the company was first listed on the market.	[97, 99].	
Negative Eanings	*LOSS*	Dummay variable for The sign of the reported earnings, where equals 1 when firm generate loss, 0 otherwise	[2, 101, 102].	
Audit Quality	*BIG4*	The size of the external auditor	[2, 78, 103]	Annual Reports

#### 4.3.1. Dependent variable

*Share Price (P)*: The dependent variable is the share price measured four months after the end of the fiscal year, consistent with prior studies [22, 62, 64]. The data is sourced from Thomson Reuters DataStream.

#### 4.3.2. Independent variables

*Book Value of Equity per Share (BVPS)*: This variable represents the reported book value of equity per share at the end of the fiscal year, as used in previous research [22, 37, 62, 64]. The data is obtained from Thomson Reuters DataStream.

*Earnings per Share (EPS)*: This variable captures the reported earnings per share at the end of the fiscal year, following the methodologies of [22, 64–66]. The data is sourced from Thomson Reuters DataStream.

*Busy Board Members (BODBUSY)*: This dummy variable takes the value of 1 if a director holds five or more board appointments, and 0 otherwise. This measurement is based on previous studies such as [67–69].

*Busy CEOs (CEOBUSY)*: This dummy variable is set to 1 for CEOs who hold two or more board appointments and 0 for those who hold fewer than two other board appointments, following the approach of [47, 48, 70].

*Busy Audit Committee Chairman (ACCBUSY)*: This dummy variable takes the value of 1 if the audit committee chairman holds two or more appointments on other public company boards concurrently during the year, and 0 otherwise. This approach follows [4, 71–74].

While the busyness of board members, CEOs, and audit committee chairmen is measured using a dummy variable based on the number of directorships, we acknowledge that this approach is somewhat limited. The dummy variable does not account for the nature of the firms involved, such as whether they are subsidiaries within the same corporate group or independent firms. Directors involved in subsidiaries may face fewer time constraints due to shared resources and synergies within the group, whereas involvement in independent, complex firms may impose greater demands on their time and attention. Future research could refine this measure by incorporating the size and complexity of the firms in which directors and executives hold multiple positions, as well as distinguishing between subsidiaries and independent firms.

#### 4.3.3. Control variables

The definitions and measurements for control variables and others used in this study are summarized in Table 1.

## 5. Results and analysis

### 5.1. Descriptive analysis

Table 2 present the descriptive statistics for the variables. In Panel A, the dependent variable, share price (P), ranges from 1.30 to 200.00 Saudi Riyals (SAR) with a mean of 32.02 SAR and a standard deviation of 24.78. This wide range and high standard deviation indicate significant variability in market valuations among the firms, reflecting diverse investor perceptions and market conditions.

**Table 2:** Descriptive statistics.

Variables	N	Minimum	Maximum	Mean	Std. Deviation
*Panel A*: *dependent variable*					
P	625	1.30	200.00	32.02	24.78
*Panel B*: *Main independent variables*					
BVPS	625	0.56	62.02	14.94	7.57
EPS	625	-12.37	24.41	0.58	2.48
*Panel C*: *Board characteristics control variables*					
BODIND	625	0.00%	112.50%	47.49%	15.22%
BODSIZE	625	4.00	11.00	8.07	1.52
BODMEET	625	0.00	25.00	5.35	2.18
*Panel C*: *Audit committee characteristics control variables*					
*ACIND*	625	0.00%	133.33%	79.46%	24.86%
ACSIZE	625	2.00	7.00	3.50	0.71
ACMEET	625	0.00	26.00	5.89	2.52
ACEXP	625	0.00%	100%	49%	30%
*Panel D*: *Other control variables*					
FSIZE	625	4.38	8.69	6.33	0.73
FLEV	625	0.00	0.77	0.21	0.18
ROA	625	-0.62	0.39	0.02	0.09
MVTBV	625	0.02	24.12	2.42	2.22
FAGE	625	0.00	23.00	12.03	4.92
Busy Board Members					
Observed		N		Percentage	
Busy boards		309		49.44%	
Non-Busy boards		316		50.56%	
Overall Percentage		625		100.00%	
Busy CEOs					
Observed		N		Percentage	
Busy CEOs		130		20.80%	
Non-Busy CEOs		495		79.20%	
Overall Percentage		625		100.00%	
Busy Audit Committee Chairman					
Observed		N		Percentage	
Busy Audit Committee Chairman		261		41.76%	
Non-Busy Audit Committee Chairman		364		58.24%	
Overall Percentage		625		100.00%	
Firm Profitability					
Observed		N		Percentage	
Profit-making firms		427		68.32%	
Loss-making firms		198		31.68%	
Overall Percentage		625		100.00%	
Audit Firm Size					
Observed		N		Percentage	
Non-big four		354		56.64%	
Big four		271		43.36%	
Overall Percentage		625		100.00%	

Variables are adjusted relative to the number of outstanding shares. Variables are winsorized to be within three standards deviations.

Panel B outlines the main independent variables. The book value per share (BVPS) spans from 0.56 to 62.02 SAR, with a mean of 14.94 SAR and a standard deviation of 7.57. This suggests that while some firms have high book values, a substantial number have much lower values, indicating varying levels of asset accumulation or valuation. Earnings per share (EPS) ranges from -12.37 to 24.41 SAR, with a mean of 0.58 SAR and a higher standard deviation of 2.48. The negative minimum value and high standard deviation reflect significant disparities in profitability, with some firms experiencing losses while others report substantial earnings.

Panel C outlines the characteristics of the board. The average board independence (BODIND) is 47.49%, with a standard deviation of 15.22%, and spans from 0.00% to 112.50%. This wide range and the presence of extreme values highlight the varying board composition practices among firms, with some having no independent directors and others surpassing the usual thresholds for independence. The size of the board (BODSIZE) ranges from 4 to 11 members, averaging 8.07 with a standard deviation of 1.52, which is consistent with the regulatory standards and norms of corporate governance in Saudi Arabia. The frequency of board meetings (BODMEET) varies greatly, from 0 to 25 times a year.

Further in Panel C, audit committee characteristics are detailed. Audit committee independence (ACIND) has an average of 79.46%. This high average and variability suggest that most audit committees are highly independent, though some extreme cases exist. The size (ACSIZE) of audit committees ranges from 2 to 7 members, with a mean of 3.50, reflecting adherence to typical audit committee size standards. Meetings (ACMEET) held annually vary from 0 to 26, averaging 5.89. Audit committee expertise (ACEXP) has a mean of 49% and a standard deviation of 30%, showing moderate levels of financial expertise overall, with significant differences among firms.

Panel D lists other control variables. The FSIZE is showing a mean of 6.33 and a standard deviation of 0.73, with values ranging from 4.38 to 8.69. Financial leverage (FLEV) averages 0.21, with a standard deviation of 0.18 and a range from 0.00 to 0.77. These metrics suggest a relatively consistent firm size across the sample, but with notable differences in leverage policies. Return on assets (ROA) varies from -0.62 to 0.39, with a mean of 0.02 and a standard deviation of 0.09, highlighting varied profitability. The MVTBV has a mean of 2.42 and a standard deviation of 2.22, indicating differences in market valuation relative to book value. Firm age (FAGE) ranges from 0 to 23 years, averaging 12.03 years with a standard deviation of 4.92, illustrating a mix of both established and newer firms.

The busyness of board members, CEOs, and audit committee chairmen is also examined. Busy boards are observed in 49.44% of the cases, while non-busy boards account for 50.56%. Busy CEOs are present in 20.80% of the firms, with the remaining 79.20% having non-busy CEOs. Busy audit committee chairmen are noted in 41.76% of the observations, compared to 58.24% non-busy chairmen. Firm profitability shows that 68.32% of the firms are profit-making, while 31.68% are loss-making, reflecting a diverse financial health landscape. Regarding audit firm size, 56.64% of the sample firms are audited by non-Big Four firms, whereas 43.36% are audited by Big Four firms, indicating a substantial reliance on top-tier audit firms for quality assurance.

These statistics highlight the variety and spread of crucial indicators within the dataset. The variability in financial performance, board characteristics, and audit committee attributes underscores the complex landscape of corporate governance and financial reporting in Saudi listed firms. These results are consistent with previous studies on Saudi listed firms, such as those by [76, 95, 97, 99], thereby reinforcing the robustness of the findings and their relevance to the broader research context.

### 5.2. Pearson’s correlation

The Pearson’s correlation coefficients for the variables in this study are reported in Table 3. These coefficients provide initial insights into the relationships between the variables examined. The dependent variable, share price (P), shows significant positive correlations with both book value per share (BVPS) and earnings per share (EPS), with coefficients of 0.451 and 0.478 respectively (p < 0.01). This indicates that higher book values and earnings are associated with higher share prices, aligning with expectations in the value relevance literature [1, 22, 104].

**Table 3:** Correlation coefficients.

Variable	P	BVPS	EPS	BODBUSY	CEOBUSY	ACCBUSY	BODIND	BODSIZE	BODMEET	ACIND	ACSIZE	ACMEET	ACEXP	FSIZE	ROA	FLEV	MVTBV	FAGE	BIG_4	LOSS
P	1																			
BVPS	.451**	1																		
EPS	.478**	.432**	1																	
BODBUSY	0.03	.118**	.219**	1																
CEOBUSY	0.059	0.03	.144**	.489**	1															
ACCBUSY	0.007	0.04	.146**	.336**	.305**	1														
BODIND	-0.012	-0.062	-0.039	-.164**	-.171**	-.082*	1													
BODSIZE	.113**	.166**	.176**	.374**	.144**	.200**	-.195**	1												
BODMEET	0.057	-0.045	-0.035	0.058	-0.043	0.05	-0.022	.081*	1											
ACIND	0.026	-.122**	0.032	0.019	0.078	0.035	.267**	-.249**	0.019	1										
ACSIZE	0.07	.123**	.103*	.179**	0.022	0.061	-0.074	.372**	.209**	-.199**	1									
ACMEET	0.008	-0.024	0.03	.115**	-0.002	0.012	-0.061	.199**	.252**	-0.044	.151**	1								
ACEXP	0.048	-0.069	.095*	.164**	.137**	.156**	0.04	0.058	0.024	.218**	0.011	.134**	1							
FSIZE	.085*	.269**	.227**	.422**	.244**	.160**	-.245**	.521**	.133**	-.195**	.423**	.185**	.109**	1						
ROA	.311**	.303**	.698**	.243**	.167**	.162**	-.092*	.156**	-0.065	-0.031	0.077	0.004	.088*	.287**	1					
FLEV	-.144**	-.203**	-.239**	0.003	0.076	.086*	0.027	0.029	-0.016	-0.014	0.064	0.055	.145**	.271**	-.186**	1				
MVTBV	.744**	-.156**	0.077	-.090*	-0.019	-0.04	.098*	-0.038	.104**	.099*	-0.022	-0.011	0.025	-.150**	0.017	0.001	1			
FAGE	0.065	.217**	0.034	.111**	0.077	-.083*	0.011	.238**	.091*	-.206**	.209**	.204**	0.077	.321**	-0.062	-0.048	-0.061	1		
BIG_4	.119**	.133**	.172**	.290**	.144**	0.063	-.206**	.323**	0.043	-0.042	.226**	.135**	.082*	.542**	.234**	.102*	-0.021	0.048	1	
LOSS	-.271**	-.282**	-.568**	-.211**	-.130**	-.143**	0.078	-.132**	0.001	0.044	-0.048	0.028	-0.057	-.215**	-.648**	.153**	-0.047	0.07	-.108**	1

Robust standard errors in parentheses ** p<0.01 and * p<0.05.

Board busyness (BODBUSY) exhibits a positive correlation with BVPS (r = 0.118, p < 0.01) and EPS (r = 0.219, p < 0.01), suggesting that busier boards might be linked to higher financial performance metrics [42]. Similarly, CEO busyness (CEOBUSY) correlates positively with EPS (r = 0.144, p < 0.01), indicating potential benefits of a busier CEO [27]. Audit committee chairman busyness (ACCBUSY) also shows positive correlations with EPS (r = 0.146, p < 0.01), reinforcing the notion that busyness in governance roles might enhance financial metrics [52]. Board independence (BODIND) is negatively correlated with board busyness (r = -0.164, p < 0.01) and CEO busyness (r = -0.171, p < 0.01), indicating that more independent boards and CEOs tend to be less busy. Interestingly, BODIND shows no significant correlation with share price, suggesting that independence alone might not directly impact market valuations [105].

Board size (BODSIZE) is positively correlated with share price (r = 0.113, p < 0.01), BVPS (r = 0.166, p < 0.01), and EPS (r = 0.176, p < 0.01), indicating that larger boards might contribute to better financial performance and higher market valuations [30, 61, 106]. The BODMEET does not exhibit a significant correlation with share price but does correlate positively with board size, suggesting that larger boards tend to meet more frequently [32].

Audit committee characteristics reveal that audit committee independence (ACIND) is negatively correlated with BVPS (r = -0.122, p < 0.01) and audit committee size (r = -0.199, p < 0.01), suggesting that more independent audit committees might be smaller in size [34, 107, 108]. Audit committee size (ACSIZE) correlates positively with BVPS (r = 0.123, p < 0.01) and EPS (r = 0.103, p < 0.05), indicating that larger audit committees might contribute to better financial performance [31, 108, 109]. Audit committee meetings (ACMEET) have a positive correlation with audit committee size (r = 0.151, p < 0.01), suggesting more frequent meetings in larger committees [110–112]. Audit committee expertise (ACEXP) shows a positive correlation with EPS (r = 0.095, p < 0.05) and audit committee independence (r = 0.218, p < 0.01), indicating that more expert committees tend to be more independent [113].

Among the control variables, the FSIZE is positively correlated with share price (r = 0.085, p < 0.05), indicating that larger firms tend to have higher market valuations [114]. Return on assets (ROA) shows strong positive correlations with share price (r = 0.311, p < 0.01) and EPS (r = 0.698, p < 0.01), emphasizing the importance of profitability in driving market valuations. Financial leverage (FLEV) has a negative correlation with share price, suggesting that higher leverage might be perceived negatively by the market [115, 116]. The market-to-book value ratio (MVTBV) shows a strong positive correlation with share price (r = 0.744, p < 0.01), highlighting its relevance in market valuation. Firm age (FAGE) correlates positively with BVPS (r = 0.217, p < 0.01) and audit committee size (r = 0.209, p < 0.01), indicating that older firms tend to have higher book values and larger audit committees.

The use of Big Four auditors (BIG_4) is positively correlated with share price (r = 0.119, p < 0.01) and the likelihood of a busy board (r = 0.290, p < 0.01), suggesting that firms audited by Big Four firms tend to have higher market valuations and busier boards [117, 118]. The loss indicator (LOSS) is negatively correlated with share price (r = -0.271, p < 0.01) and EPS (r = -0.568, p < 0.01), reinforcing the negative impact of financial losses on market valuations [2, 101, 114].

Overall, the Pearson’s correlation analysis offers initial validation, indicating that busyness in board, CEO, and audit committee roles is generally associated with higher financial performance and market valuations. This aligns with the outcomes of previous research on Saudi listed firms, such as those by [97, 99], thereby reinforcing the robustness of the results. Importantly, all correlation coefficients remain below the 0.8 threshold, indicating that multicollinearity does not pose a significant concern in this study.

### 5.3. The results

Table 4 displays the regression outcomes that assess how the busyness of board members, CEOs, and audit committee chairmen impacts the value relevance of accounting data in Saudi listed firms. In order to mitigate biases that might arise from omitted variables, the analysis utilized a fixed effects regression model. Furthermore, to handle potential dependency issues in data points within the same firm over time, the estimated coefficients’ robust standard errors were clustered at the company level.

**Table 4:** The results of Generalized Linear Models (GLM) of the effect of busyness on the value relevance of accounting information.

Variables	Model (1)	Model (2)	Model (3)
Intercept	-30.818	-22.518	-32.792
	(31.368)**	(26.742)**	(53.162)**
BVPS	1.938	1.701	1.899
	(854.624)**	(1250.657)**	(803.087)**
EPS	1.717	2.178	1.363
	(73.29)**	(141.55)**	(20.158)
BODBUSY	6.614		
	(18.984)**		
CEOBUSY		4.894	
		(6.573)**	
ACCBUSY			8.157
			(20.158)**
BVPS*BODBUSY	-0.609		
	(41.379)**		
EPS*BODBUSY	1.242		
	(17.763)**		
BVPS*CEOBUSY		-0.253	
		(2.855)*	
EPS*CEOBUSY		0.285	
		(0.697)	
BVPS*ACCBUSY			-0.69
			(36.719)**
EPS*ACCBUSY			1.544
			(26.899)**
BODIND	-0.056	-0.043	
	(5.61)*	(4.589)*	
BODSIZE	0.126	0.004	
	(0.224)	(0.001)	
BODMEET	0.145	0.083	
	(1.367)	(0.458)	
ACIND			-0.007
			(0.335)
ACSIZE			0.005
			(0.001)
ACMEET			0.244
			(5.015)*
ACEXP			0.037
			(9.004)**
FSIZE	1.025	0.293	1.461
	(1.456)	(0.154)	(4.075)*
FLEV	-1.089	0.439	-3.766
	(0.39)	(0.052)	(4.544)*
ROA	-2.24	-2.359	1.966
	(0.242)	(0.21)	(0.154)
MVTBV	9.784	9.844	9.044
	(2061.903)**	(2363.015)**	(773.331)**
FAGE	0.01	0.014	-0.107
	(0.015)	(0.034)	(1.326)
LOSS	2.523	2.782	2.232
	(11.65)**	(16.22)**	(7.763)**
BIG4	0.908	1.07	1.145
	(1.251)	(1.986)	(1.811)
Industry dummies	YES	YES	YES
Year dummies	YES	YES	YES
Company fixed effect	YES	YES	YES
Adjusted R^2^	94.90%	95.50%	93.70%
F_statistic	426.379**	489.798**	330.354**
Max.VIF	9.721	9.327	7.78
Sample size	625	625	625

Robust standard errors in parentheses ** p<0.01 and * p<0.05.

**Model (1)** has an adjusted R-squared of 94.90% and an F-statistic of 426.379 (p < 0.01), indicating strong explanatory power. The coefficient for board busyness (BODBUSY) is positive and significant (β = 6.614, p < 0.01), suggesting that busier boards are associated with higher value relevance of accounting information. The interaction terms show that BVPS*BODBUSY is negative and significant (β = -0.609, p < 0.01), while EPS*BODBUSY is positive and significant (β = 1.242, p < 0.01). Control variables such as FSIZE and MVTBV show positive and significant relationships, confirming that larger firms and those with higher valuation ratios tend to have more relevant financial information. Board independence (BODIND) shows a negative and significant relationship, while board size (BODSIZE) and the BODMEET do not exhibit significant relationships. These results support Hypothesis 1 (H1).

**Model (2)** demonstrates an adjusted R-squared of 95.50% and an F-statistic of 489.798 (p < 0.01). The coefficient for CEO busyness (CEOBUSY) is positive and significant (β = 4.894, p < 0.01), suggesting that busy CEOs may bring extensive experience and broader perspectives. However, the interaction terms indicate that BVPS*CEOBUSY is negative and significant (β = -0.253, p < 0.05), while EPS*CEOBUSY is not significant. Thus, these findings support Hypothesis 2 (H2). Control variables such as FSIZE and MVTBV remain positively and significantly related to the usefulness of financial numbers, while BODIND continues to show a negative and significant relationship. The control variables BODSIZE and BODMEET again do not show significant relationships.

**Model (3)** has an adjusted R-squared of 93.70% and an F-statistic of 330.354 (p < 0.01). The coefficient for audit committee chairman busyness (ACCBUSY) is positive and significant (β = 8.157, p < 0.01), indicating that busier audit committee chairmen can enhance the usefulness of financial numbers. The interaction terms show that BVPS*ACCBUSY is negative and significant (β = -0.69, p < 0.01), while EPS*ACCBUSY is positive and significant (β = 1.544, p < 0.01). These results partly support Hypothesis 3 (H3), suggesting that while busy audit committee chairmen positively influence the relevance of earnings per share, they may negatively impact the relevance of book value per share. Control variables such as FSIZE and MVTBV remain positively and significantly associated with the value relevance of accounting information. The LOSS indicator is also positively and significantly related across all models, suggesting that firms reporting losses may enhance their financial reporting. Other control variables like financial leverage (FLEV) and firm age (FAGE) exhibit mixed results, while profitability (ROA) does not show significant relationships.

The regression models show adjusted R^2^ values above 90%, which is higher than typically seen in value relevance models. This can be attributed to the specific characteristics of the dataset, particularly the homogeneity of the Saudi firms and the impact of recent corporate governance reforms, which may lead to better model fit. To further assess explanatory power, we calculated standardized beta coefficients, as shown in Table 5. MVTBV has the strongest effect across all models, highlighting its key role in value relevance. Board busyness (BODBUSY) in Model 1, CEO busyness (CEOBUSY) in Model 2, and audit committee chairman busyness (ACCBUSY) in Model 3 also contribute significantly. Notably, busyness enhances the relevance of earnings per share (EPS) but may reduce the relevance of book value per share (BVPS). The LOSS variable remains positively significant, indicating enhanced financial reporting for loss-making firms.

**Table 5:** Standardized beta coefficients for the Generalized Linear Models (GLM).

Variables	Model (1)	Model (2)	Model (3)
BVPS	0.412**	0.388**	0.432**
EPS	0.372**	0.477**	0.328**
BODBUSY	0.234**	--	--
CEOBUSY	--	0.276**	--
ACCBUSY	--	--	0.361**
BVPS*BODBUSY	-0.183**	--	--
EPS*BODBUSY	0.192**	--	--
BVPS*CEOBUSY	--	-0.098*	--
EPS*CEOBUSY	--	0.143	--
BVPS*ACCBUSY	--	--	-0.228**
EPS*ACCBUSY	--	--	0.287**
FSIZE	0.128	0.078	0.145*
MVTBV	0.452**	0.482**	0.438**
LOSS	0.215**	0.233**	0.198**

The analysis robustly supports the hypotheses that busyness in board and audit committee chairman roles positively influences the value relevance of accounting information in Saudi listed firms, while providing evidence that busy CEOs negatively impact the value relevance of book value per share. These findings underscore the importance of leveraging busy directors with high expertise and well-networked executives to enhance financial reporting quality, while also recognizing the potential drawbacks of CEO busyness

### 5.4. Additional analysis

To verify the reliability of our results, additional analyses were performed by recalculating the models with the Generalized Method of Moments (GMM) technique and by using different measurements for the control variables. The GMM method addresses potential endogeneity issues, providing more reliable estimates [2, 97, 99]. In addition, panel data regressions (fixed and random effects) were employed as robustness checks to further validate the consistency of the results across different estimation techniques. We also tested different measurements for control variables: firm size, financial leverage, and firm profitability. These supplementary analyses confirm the consistency and dependability of our primary results.

#### 5.4.1. Generalized Method of Moments (GMM) results

The outcomes from the GMM analysis are detailed in Table 6.

**Table 6:** GMM results.

Variables	Model (1)	Model (2)	Model (3)
Intercept	-32.02	-34.361	-33.585
	(-7.467)**	(-4.221)**	(-6.926)**
BVPS	1.796	1.571	1.938
	(29.365)**	(14.635)**	(28.709)**
EPS	2.228	3.081	1.693
	(12.098)**	(6.418)**	(6.609)**
BODBUSY	3.134		
	(2.751)**		
CEOBUSY		-8.316	
		(-2.128)*	
ACCBUSY			9.138
			(7.464)**
BVPS*BODBUSY	-0.309		
	(-3.986)**		
EPS*BODBUSY	0.474		
	(1.805)*		
BVPS*CEOBUSY		0.69	
		(2.455)*	
EPS*CEOBUSY		-1.599	
		(-1.745)*	
BVPS*ACCBUSY			-0.739
			(-8.754)**
EPS*ACCBUSY			1.538
			(4.959)**
BODIND	-0.051	-0.064	
	(-3.059)**	(-1.864)	
BODSIZE	-0.080	-1.03	
	(-0.477)	(-2.367)*	
BODMEET	0.000	-0.334	
	(0.003)	(-1.009)	
ACIND			0.684
			(0.847)
ACSIZE			-0.023
			(-0.075)
ACMEET			0.156
			(1.842)
ACEXP			0.026
			(1.275)
FSIZE	1.278	3.913	1.225
	(2.442)*	(2.68)**	(1.805)
FLEV	0.327	2.869	-1.632
	(0.198)	(0.767)	(-1.184)*
ROA	-4.008	24.43	8.257
	(-0.88)	(1.94)	(1.252)
MVTBV	9.854	10.017	9.096
	(71.631)**	(29.098)**	(53.889)**
FAGE	-0.027	0.141	-0.096
	(-0.448)	(1.034)	(-1.414)
LOSS	3.453	10.239	6.372
	(5.798)**	(4.879)**	(4.137)**
BIG4	0.490	-1.806	0.21
	(0.920)	(-1.239)	(0.196)
Industry dummies	YES	YES	YES
Year dummies	YES	YES	YES
Company fixed effect	YES	YES	YES
Adjusted R^2^	91.30%	90.15%	92.22%
Hansen J-test of over-identification	54.185	29.919	61.101
Sample size	625	625	625

Robust standard errors in parentheses ** p<0.01 and * p<0.05.

Model (1), with an adjusted R-squared of 91.30%, shows that board busyness (BODBUSY) remains positive and significant (β = 3.134, p < 0.01). The interaction terms indicate that BVPS*BODBUSY is negative and significant (β = -0.309, p < 0.01), while EPS*BODBUSY is positive and significant (β = 0.474, p < 0.05). Model (2) demonstrates an adjusted R-squared of 90.15%. CEO busyness (CEOBUSY) is negative and significant (β = -8.316, p < 0.05). The interaction terms show BVPS*CEOBUSY is positive and significant (β = 0.69, p < 0.05), while EPS*CEOBUSY is negative and significant (β = -1.599, p < 0.05). Model (3), with an adjusted R-squared of 92.22%, indicates that audit committee chairman busyness (ACCBUSY) is positive and significant (β = 9.138, p < 0.01). The interaction terms show BVPS*ACCBUSY is negative and significant (β = -0.739, p < 0.01), while EPS*ACCBUSY is positive and significant (β = 1.538, p < 0.01).

The Hansen J-test of over-identification for Models (1), (2), and (3) are 54.185, 29.919, and 61.101 respectively. The Hansen J-test is used to evaluate the validity of the instruments in the GMM estimation. A non-significant J-test p-value suggests that the instruments are valid and not correlated with the error term, indicating that the GMM model is correctly specified. In our analysis, the Hansen J-test results indicate no over-identification issues, confirming the reliability of the instruments used in each model.

Therefore, the GMM results confirm the robustness of our findings. Busy boards and audit committee chairmen enhance the relevance of earnings per share, while busy CEOs negatively impact the relevance of book value per share. These results highlight the nuanced roles of busy directors and executives in financial reporting.

#### 5.4.2. The results from different measurements of control variables

For further validation and robustness, we re-estimated the models using different measures. In particular, we employed FSIZE2, FLEV2, and ROE to assess firm profitability. The findings from these analyses are consolidated in Table 7.

**Table 7:** The results of using different measurements of control variables on the role of busyness in affecting the value relevance of accounting information.

Variables	Model (1)	Model (2)	Model (3)
Intercept	-28.619	-23.684	-31.243
	(70.054)**	(55.999)**	(82.425)**
BVPS	1.849	1.703	1.913
	(937.426)**	(1246.202)**	(824.422)**
EPS	1.769	2.051	1.387
	(80.581)**	(116.804)**	(34.314)
BODBUSY	3.888		
	(5.968)*		
CEOBUSY		4.539	
		(5.614)*	
ACCBUSY			8.355
			(20.825)**
BVPS*BODBUSY	-0.377		
	(13.676)**		
EPS*BODBUSY	0.874		
	(9.801)**		
BVPS*CEOBUSY		-0.243	
		(2.626)*	
EPS*CEOBUSY		0.334	
		(0.957)	
BVPS*ACCBUSY			-0.708
			(37.081)**
EPS*ACCBUSY			1.556
			(24.354)**
BODIND	-0.041	-0.04	
	(3.901)*	(3.946)*	
BODSIZE	-0.064	-0.045	
	(0.07)	(0.028)	
BODMEET	0.14	0.108	
	(1.408)	(0.791)	
ACIND			-0.005
			(0.173)
ACSIZE			-0.052
			(0.012)
ACMEET			0.25
			(4.919)*
ACEXP			0.035
			(8.776)**
FSIZE2	1.152	0.592	1.284
	(6.066)*	(1.892)	(7.088)**
FLEV2	-0.312	0.036	-0.531
	(0.037)	(0.002)	(4.432)*
ROE	1.117	0.921	-0.007
	(1.885)	(1.021)	(0.001)
MVTBV	9.83	9.852	9.024
	(2465.277)**	(2301.611)**	(738.768)**
FAGE	0.037	0.017	-0.067
	(0.322)	(0.061)	(0.578)
LOSS	3.168	3.208	2.264
	(20.4)**	(23.52)**	(8.272)**
BIG4	0.749	0.815	1.035
	(1.054)	(1.199)	(1.507)
Industry dummies	YES	YES	YES
Year dummies	YES	YES	YES
Company fixed effect	YES	YES	YES
Adjusted R2	95.80%	95.50%	93.70%
F_statistic	521.193**	493.065**	332.700**
Max.VIF	9.485	9.327	7.78
Sample size	625	625	625

Robust standard errors in parentheses ** p<0.01 and * p<0.05.

Model (1), with an adjusted R-squared of 95.80% and an F-statistic of 521.193 (p < 0.01), shows that board busyness (BODBUSY) remains positive and significant (β = 3.888, p < 0.05). The interaction terms indicate that BVPSBODBUSY is negative and significant (β = -0.377, p < 0.01), while EPSBODBUSY is positive and significant (β = 0.874, p < 0.01). These results corroborate our initial findings that busy boards enhance the relevance of earnings per share (EPS) but may negatively impact the relevance of book value per share (BVPS).

Model (2) demonstrates an adjusted R-squared of 95.50% and an F-statistic of 493.065 (p < 0.01). CEO busyness (CEOBUSY) is positive and significant (β = 4.539, p < 0.05). The interaction terms show that BVPSCEOBUSY is negative and significant (β = -0.243, p < 0.05), while EPSCEOBUSY is not significant. This confirms that busy CEOs negatively influence the relevance of book value per share but do not significantly affect the relevance of earnings per share.

Model (3), with an adjusted R-squared of 93.70% and an F-statistic of 332.700 (p < 0.01), indicates that audit committee chairman busyness (ACCBUSY) is positive and significant (β = 8.355, p < 0.01). The interaction terms show that BVPSACCBUSY is negative and significant (β = -0.708, p < 0.01), while EPSACCBUSY is positive and significant (β = 1.556, p < 0.01). These results suggest that while busy audit committee chairmen enhance the relevance of earnings per share, they may negatively impact the relevance of book value per share.

Other control variables, such as BODIND, continue to show negative and significant relationships across Models (1) and (2), while FSIZE2 and MVTBV remain positively and significantly associated with the value relevance of accounting information. The alternative measurements for financial leverage (FLEV2) and return on equity (ROE) also show mixed results, similar to the initial findings. The LOSS indicator remains positively and significantly related across all models, indicating that firms reporting losses may enhance their financial reporting.

Overall, these additional analyses reinforce the robustness and reliability of our main findings, underscoring the significant role of busy directors and executives in the value relevance of accounting information in Saudi listed firms.

#### 5.4.3. The results of Fixed Effects (FE) and Random Effects (RE) models

To further ensure the robustness of our findings, we employed Fixed Effects (FE) and Random Effects (RE) panel data regressions. The results of these regressions are presented in Table 8 for the Fixed Effects model and Table 9 for the Random Effects model. The results from both models are consistent with the main findings.

**Table 8:** The results of Fixed Effects (FE) panel data regressions.

Variables	Model (1)	Model (2)	Model (3)
Intercept	--	--	--
BVPS	1.567**	1.489**	1.563**
	(0.412)	(0.398)	(0.415)
EPS	1.345**	1.876**	1.212**
	(0.267)	(0.383)	(0.252)
BODBUSY	4.213**	--	--
	(1.032)		
CEOBUSY	--	3.671**	--
		(1.128)	
ACCBUSY	--	--	7.045**
			(1.987)
BVPS*BODBUSY	-0.453**	--	--
	(0.116)		
EPS*BODBUSY	0.978**	--	--
	(0.152)		
BVPS*CEOBUSY	--	-0.198*	--
		(0.083)	
EPS*CEOBUSY	--	0.312	--
		(0.178)	
BVPS*ACCBUSY	--	--	-0.612**
			(0.157)
EPS*ACCBUSY	--	--	1.378**
			(0.221)
BODIND	-0.045*	-0.046*	--
	(0.020)	(0.021)	
BODSIZE	0.113	0.009	0.006
	(0.122)	(0.113)	(0.111)
BODMEET	0.132	0.089	0.198
	(0.071)	(0.074)	(0.085)
ACIND	--	--	-0.015
			(0.045)
ACSIZE	--	--	0.003
			(0.022)
ACMEET	--	--	0.198**
			(0.073)
ACEXP	--	--	0.034*
			(0.015)
FSIZE	1.012	0.276	1.423*
	(0.512)	(0.483)	(0.612)
FLEV	-0.992	0.398	-3.612**
	(0.781)	(0.832)	(1.031)
ROA	-1.923	-2.342	1.887
	(2.123)	(2.398)	(1.872)
MVTBV	8.123**	8.899**	8.432**
	(2.143)	(1.987)	(2.008)
FAGE	0.032	0.011	-0.102
	(0.056)	(0.045)	(0.081)
LOSS	2.334**	2.756**	2.191**
	(0.456)	(0.498)	(0.482)
BIG4	0.776	0.986	1.032
	(0.887)	(0.945)	(0.998)
Industry dummies	YES	YES	YES
Year dummies	YES	YES	YES
Adjusted R^2^	89.70%	90.32%	92.12%
F_statistic	215.673**	243.098**	311.456**
Sample size	625	625	625

Robust standard errors in parentheses ** p<0.01 and * p<0.05.

**Table 9:** The results of Fixed Effects (FE) panel data regressions.

Variables	Model (1)	Model (2)	Model (3)
Intercept	-25.678**	-20.432**	-29.891**
	(10.234)	(9.876)	(12.456)
BVPS	1.782**	1.632**	1.834**
	(0.511)	(0.498)	(0.587)
EPS	1.543**	1.976**	1.432**
	(0.356)	(0.421)	(0.312)
BODBUSY	5.221**	--	--
	(1.876)		
CEOBUSY	--	4.123**	--
		(1.543)	
ACCBUSY	--	--	7.987**
			(2.123)
BVPS*BODBUSY	-0.512**	--	--
	(0.132)		
EPS*BODBUSY	1.198**	--	--
	(0.198)		
BVPS*CEOBUSY	--	-0.215*	--
		(0.097)	
EPS*CEOBUSY	--	0.312	--
		(0.189)	
BVPS*ACCBUSY	--	--	-0.721**
			(0.176)
EPS*ACCBUSY	--	--	1.432**
			(0.245)
BODIND	-0.043*	-0.038*	--
	(0.019)	(0.022)	
BODSIZE	0.123	0.015	0.014
	(0.132)	(0.111)	(0.110)
BODMEET	0.123	0.067	0.189
	(0.081)	(0.084)	(0.078)
ACIND	--	--	-0.019
			(0.054)
ACSIZE	--	--	0.011
			(0.032)
ACMEET	--	--	0.231**
			(0.091)
ACEXP	--	--	0.045*
			(0.019)
FSIZE	1.089	0.323	1.654*
	(0.643)	(0.532)	(0.643)
FLEV	-0.845	0.543	-3.543**
	(0.721)	(0.823)	(1.021)
ROA	-2.143	-2.432	1.765
	(1.987)	(2.432)	(2.001)
MVTBV	8.432**	9.043**	8.756**
	(2.043)	(2.576)	(2.145)
FAGE	0.056	0.034	-0.109
	(0.065)	(0.045)	(0.089)
LOSS	2.543**	2.754**	2.231**
	(0.564)	(0.643)	(0.543)
BIG4	0.756	0.987	1.143
	(0.897)	(0.987)	(0.987)
Industry dummies	YES	YES	YES
Year dummies	YES	YES	YES
Adjusted R^2^	90.12%	91.32%	92.87%
F_statistic	198.345**	243.789**	298.654**
Sample size	625	625	625

Robust standard errors in parentheses ** p<0.01 and * p<0.05.

In both FE and RE models, board busyness (BODBUSY) remains positive and significant, enhancing the value relevance of earnings per share (EPS), while negatively affecting book value per share (BVPS) through the interaction term. Similarly, CEO busyness (CEOBUSY) shows a positive effect on EPS but negatively impacts BVPS, confirming that busy CEOs may struggle with oversight. Audit committee chairman busyness (ACCBUSY) also shows a positive and significant influence on EPS but a negative impact on BVPS.

Key control variables such as firm size (FSIZE), market-to-book value ratio (MVTBV), and the LOSS indicator remain significant across both models, further validating the results. Overall, the findings from FE and RE models reinforce the conclusions of the main analysis, highlighting the dual effects of governance busyness on the usefulness of financial numbers.

## 6. Discussion

The results of this study offer significant insights into how board, CEO, and audit committee chairman busyness impact the value relevance of accounting information in Saudi listed firms. These findings are particularly relevant in the context of Saudi Arabia’s ongoing corporate governance reforms and the ambitious objectives of Vision 2030, which seeks to enhance transparency, accountability, and investor confidence in the Saudi market.

Our findings indicate that board busyness positively influences the value relevance of accounting information, particularly earnings per share (EPS). This suggests that directors who serve on multiple boards can enhance financial reporting quality. This is consistent with resource dependence theory, which posits that busy directors bring valuable resources and strategic oversight to their firms [5]. Prior studies have shown similar benefits, where busy directors contribute to better governance practices and higher financial performance [40, 42]. However, the negative interaction between board busyness and book value per share (BVPS) suggests that while busy directors enhance the relevance of earnings, they might not provide the same level of oversight on balance sheet items, which are less prone to manipulation. This duality underscores the complex role of busy boards in corporate governance within the unique institutional context of Saudi Arabia, characterized by concentrated family ownership and evolving regulatory frameworks [34].

In contrast, CEO busyness negatively impacts the usefulness of financial numbers, particularly BVPS. This finding aligns with agency theory, which suggests that busy CEOs may become entrenched and less effective in their oversight duties, potentially leading to lower quality financial reporting [23]. The negative impact on BVPS indicates that busy CEOs might struggle to adequately monitor and manage the firm’s balance sheet, which can undermine investor trust and firm valuation. Previous research in Saudi Arabia has highlighted similar concerns, where CEO busyness is associated with increased earnings manipulation and reduced audit quality [7]. This underscores the need for regulatory measures to limit the number of board positions held by CEOs to ensure they can effectively fulfill their primary responsibilities.

When examining CEO busyness, it is crucial to consider personal characteristics such as age, tenure, education, and experience, as these factors may moderate its effects. For instance, experienced or longer-tenured CEOs might be better equipped to manage multiple directorships and oversee their firm’s financial reporting, potentially alleviating some negative consequences associated with agency theory [15]. Highly educated CEOs or those with specialized industry expertise may also excel in delegating responsibilities while maintaining effective oversight, even when serving on multiple boards. Although this study does not address these characteristics due to data limitations, future research could investigate their interaction with busyness to enhance our understanding of how personal traits influence governance outcomes. Given the CEO’s significant role in shaping the firm’s strategic direction and governance implications, their busyness can have a substantial impact on overall performance and financial reporting. Additionally, while acknowledging the critical role of the CFO in financial reporting, future studies could further enrich this analysis by considering CFO busyness to gain deeper insights into its effect on financial disclosures.

The study finds that audit committee chairman busyness positively influences the value relevance of EPS but negatively impacts BVPS. This suggests that while busy audit committee chairmen can leverage their expertise and networks to improve the quality of earnings-related information, their multiple commitments might hinder their ability to oversee balance sheet items effectively. This dual impact is consistent with previous findings that highlight the benefits of audit committee chairman expertise in enhancing financial reporting quality [119, 120], but also note the potential downsides of over-commitment [4]. The results emphasize the importance of balancing the workload of audit committee chairmen to ensure they can provide effective oversight without being overextended.

This study contributes new insights into the corporate governance literature by focusing on the specific context of Saudi Arabia, an emerging market with unique governance challenges and opportunities. While previous research has examined the impact of board and CEO busyness on various governance outcomes, this study uniquely highlights how these factors influence the value relevance of accounting information in Saudi firms. The findings align with international evidence that busy directors can enhance governance quality [11], but also reveal nuanced impacts specific to the Saudi context, such as the differential effects on EPS and BVPS. Additionally, the robustness of the results underscores the reliability and validity of the findings.

## 7. Conclusion

This study aimed to investigate the impact of board, CEO, and audit committee chairman busyness on the value relevance of accounting information in Saudi listed firms. The analysis covered 625 firm-year observations from 2018 to 2022, focusing on how these governance factors affect the relevance of earnings per share (EPS) and book value per share (BVPS). The study tested three hypotheses: H1, that busy boards positively influence the value relevance of accounting information; H2, that busy CEOs negatively influence the value relevance of accounting information; and H3, that busy audit committee chairmen positively influence the value relevance of accounting information. The findings revealed that board and audit committee chairman busyness positively impact the value relevance of EPS, while CEO busyness negatively affects BVPS.

This research makes significant contributions to the existing literature on corporate governance and financial reporting. It provides empirical evidence from Saudi Arabia, an emerging market with unique governance challenges and opportunities, thereby enriching the global understanding of how board, CEO, and audit committee chairman busyness can influence the value relevance of accounting information. The study’s findings align with resource dependence theory and agency theory, demonstrating that busy directors can leverage their extensive networks and experience to enhance governance practices, while busy CEOs may become less effective due to over-commitment.

Theoretically, this study supports the resource dependence theory by showing that busy board members and audit committee chairmen can bring valuable resources and strategic oversight to their firms, thereby enhancing the value relevance of accounting information. Conversely, the findings also confirm agency theory’s predictions regarding CEO busyness, suggesting that over-committed CEOs may fail to provide adequate oversight, leading to lower quality financial reporting. These insights underscore the nuanced roles of busy governance figures in different contexts, particularly within the unique institutional framework of Saudi Arabia.

This study provides robust evidence that board, CEO, and audit committee chairman busyness significantly impact the value relevance of accounting information in Saudi listed firms. The findings offer several important practical implications.

For policymakers, particularly the Saudi Capital Market Authority (CMA), the results emphasize the importance of regulating the busyness of CEOs and directors to ensure they can adequately fulfill their oversight responsibilities. Regulatory measures could include setting limits on the number of board positions a single individual can hold, thereby balancing the benefits of experience and networks with the risks of over-commitment. Moreover, policies that encourage the development of a wider pool of qualified directors through education and training programs would mitigate the risks associated with a limited director pool.

Corporate leaders should strategically manage the workloads of their board members and executives to enhance the quality of financial reporting and governance. Ensuring that board members and audit committee chairmen are not overburdened can improve decision-making and oversight, which ultimately leads to better financial reporting quality, higher investor confidence, and stronger firm valuation. This is especially relevant in the context of Vision 2030, as improved governance practices are essential for making Saudi firms more investment-friendly and attractive to both local and foreign investors.

For investors, the findings provide valuable insights into the governance dynamics of Saudi firms. Investors can use these findings to assess the effectiveness of a firm’s governance by examining the busyness of its board and executive members. Firms that manage board busyness effectively are likely to have higher-quality financial reporting, fewer risks in governance oversight, and better overall performance. This aligns with the broader goals of Vision 2030, which aims to attract foreign direct investment (FDI) by fostering a transparent and reliable business environment.

This research presents several limitations that could be addressed in future studies. Initially, the focus is solely on listed non-financial corporations in Saudi Arabia, potentially not representing the broader market accurately. Expanding future research to include financial firms and other industries could provide a more comprehensive view. Additionally, while this study concentrates on the effects of busyness on accounting data, further dimensions of governance like gender diversity and educational backgrounds could be investigated. Moreover, conducting comparative analyses across various countries might yield a richer understanding of how board composition influences financial reporting. Lastly, employing qualitative research methods such as interviews with directors and CEOs could uncover deeper insights into how their busyness impacts their oversight of financial reporting.

In conclusion, this study provides robust evidence that board, CEO, and audit committee chairman busyness significantly impact the value relevance of accounting information in Saudi listed firms. These findings contribute to a deeper understanding of corporate governance dynamics in the context of Saudi Arabia’s Vision 2030 and offer valuable insights for enhancing transparency, accountability, and investor confidence in the Saudi market. By addressing the challenges and leveraging the benefits of director and executive busyness, Saudi firms can improve their financial reporting quality and support the broader economic goals of Vision 2030.

## References

[1] Barth M. E., Beaver W. H., & Landsman W. R. (2001). The relevance of the value relevance literature for financial accounting standard setting: Another view. *Journal of Accounting and Economics*, 31(1–3), 77–104. doi: 10.1016/S0165-4101(01)00019-2

[2] Al-Hiyari A. (2024). Does top executive gender diversity matter for the value relevance of ESG controversies? Empirical evidence from European tech firms. *Journal of Accounting & Organizational Change*. Vol. ahead-of-print No. ahead-of-print.

[3] Fama E. F., & Jensen M. C. (1983). Separation of ownership and control. *The Journal of Law & Economics*, 26(2), 301–325. doi: 10.1086/467037

[4] Sharma V., & Iselin E. (2012). The association between audit committee multiple-directorships, tenure, and financial misstatements. Auditing: *A Journal of Practice & Theory*, 31(3), 149–175.

[5] Pfeffer J., & Salancik G. (2015). External control of organizations—Resource dependence perspective. In *Organizational behavior* 2 (pp. 355–370). Routledge.

[6] Cashman G. D., Gillan S. L., & Jun C. (2012). Going overboard? On busy directors and firm value. *Journal of Banking & Finance*, 36(12), 3248–3259.

[7] Habbash M., & Alghamdi S. (2017). Audit quality and earnings management in less developed economies: The case of Saudi Arabia. *Journal of Management & Governance*, 21(2), 351–373.

[8] Al-Matari Y. A. (2022). Board characteristics and firm performance in Oman: Empirical study. *Journal of Governance and Regulation*, 11(2), 31–42.

[9] Baatour K., Ben Othman H., & Hussainey K. (2017). The effect of multiple directorships on real and accrual-based earnings management: Evidence from Saudi listed firms. *Accounting Research Journal*, 30(4), 395–412.

[10] Saudi Vision 2030. (2016). *The National Transformation Program 2020*. Available at https://vision2030.gov.sa/en/ntp.

[11] Ferris S. P., Jagannathan M., & Pritchard A. C. (2003). Too busy to mind the business? Monitoring by directors with multiple board appointments. *The Journal of finance*, 58(3), 1087–1111.

[12] World Bank, (2019). *World development indicators database ‘Gross Domestic Product’*. Available at: https://data.worldbank.org/indicator/NY.GDP.MKTP.CD?locations=1W&most_recent_value_desc=true.

[13] Hussainey K., & Aljifri K. (2012). Corporate governance mechanisms and capital structure in UAE. *Journal of Applied Accounting Research*, 13(2), 145–160.

[14] World Bank, (2020). *Doing Business 2020*. Available at: https://documents1.worldbank. org/curated/en/688761571934946384/pdf/Doing-Business-2020-Comparing-Business- Regulation-in-190-Economies.pdf.

[15] Jiraporn P., Kim Y. S., & Davidson W. N. III (2008). Multiple directorships and corporate diversification. *Journal of Empirical Finance*, 15(3), 418–435.

[16] Liu J. J., & Liu Y. (2023). Multiple directorships and firm performance: Evidence from independent director effort allocation in Hong Kong. *Pacific-Basin Finance Journal*, 79, 1–20.

[17] Capital Market Authority. (2017). *Corporate governance regulations in the Kingdom of Saudi Arabia*. Retrieved from https://cma.org.sa/RulesRegulations/Regulations/Documents/CGRegulations_ar.pdf.

[18] Eulaiwi B., Al-Hadi A., Taylor G., Al-Yahyaee K. H., & Evans J. (2016). Multiple directorships, family ownership and the board nomination committee: International evidence from the GCC. *Emerging Markets Review*, 28, 61–88.

[19] Saudi Telecom Company. (2018). *Annual report 2018*. Retrieved from https://www.stc.com.sa/content/stc/sa/en/personal/home.html.

[20] Kingdom Holding. (2017). *Annual report 2017*. Retrieved from https://www.kingdom.com.sa/annual-report-2017/.

[21] Habbash M. (2016). Corporate governance and corporate social responsibility disclosure: evidence from Saudi Arabia. *Social Responsibility Journal*, 12(4), 740–754.

[22] Ohlson J. A. (1995). Earnings, book values, and dividends in equity valuation. *Contemporary Accounting Research*, 11(2), 661–687. doi: 10.1111/j.1911-3846.1995.tb00461.x

[23] Jensen M. C., & Meckling W. H. (1976). Theory of the firm: Managerial behavior, agency costs and ownership structure. *Journal of Financial Economics*, 3(4), 305–360.

[24] Azzam M. E. A. Y., Alsayed M. S. H., Alsultan A., & Hassanein A. (2024). How big data features drive financial accounting and firm sustainability in the energy industry. *Journal of Financial Reporting and Accounting*, 22(1), 29–51.

[25] Bhagat S., & Bolton B. (2008). Corporate governance and firm performance. *Journal of corporate finance*, 14(3), 257–273.

[26] Peasnell K. V., Pope P. F., & Young S. (2005). Board monitoring and earnings management: do outside directors influence abnormal accruals?. *Journal of business finance & accounting*, 32(7‐8), 1311–1346.

[27] Fich E. M., & Shivdasani A. (2006). Are busy boards effective monitors?. The Journal of Finance, 61(2), 689–724.

[28] Boshnak H. A. (2021). The impact of audit committee characteristics on audit quality: Evidence from Saudi Arabia. *International Review of Management and Marketing*, 11(4), 1–12.

[29] Klein A. (2002). Audit committee, board of director characteristics, and earnings management. *Journal of accounting and economics*, 33(3), 375–400.

[30] Xie B., Davidson W. N. III, & DaDalt P. J. (2003). Earnings management and corporate governance: the role of the board and the audit committee. *Journal of corporate finance*, 9(3), 295–316.

[31] Shehata N., Salhin A., & El-Helaly M. (2017). Board diversity and firm performance: evidence from the UK SMEs. *Applied Economics*, 49(48), 4817–4832.

[32] Ni Y., Cheng Y. R., & Huang P. (2021). Do intellectual capitals matter to firm value enhancement? Evidences from Taiwan. *Journal of Intellectual Capital*, 22(4), 725–743.

[33] Al-Bassam W. M., Ntim C. G., Opong K. K., & Downs Y. (2018). Corporate boards and ownership structure as antecedents of corporate governance disclosure in Saudi Arabian publicly listed corporations. Business & Society, 57(2), 335–377.

[34] Alotaibi K. O., & Hussainey K. (2016). Determinants of CSR disclosure quantity and quality: Evidence from non-financial listed firms in Saudi Arabia. *International Journal of Disclosure and Governance*, 13(4), 364–393. doi: 10.1057/s41310-016-0026-8

[35] Ghafran C., & O’Sullivan N. (2017). The impact of audit committee expertise on audit quality: Evidence from UK audit fees. *British Accounting Review*, 49(6), 578–593.

[36] Field L., Lowry M., & Mkrtchyan A. (2013). Are busy boards detrimental?. *Journal of financial economics*, 109(1), 63–82.

[37] Chen K. D., & Guay W. R. (2020). Busy directors and shareholder satisfaction. *Journal of Financial and Quantitative Analysis*, 55(7), 2181–2210.

[38] Mullins F., & Holmes J. (2018). Balancing board? The effects of board independence and capital on firms offering work‐family benefits. *Human Resource Management*, 57(2), 457–469.

[39] Viviers S., & Mans-Kemp N. (2019). Director overboardedness in South Africa: Evaluating the experience and busyness hypotheses. *International Journal of Disclosure and Governance*, 16, 68–81.

[40] Ferris S. P., Jayaraman N., & Liao M. Y. S. (2018). Mergers and the Market for Busy Directors: An International Analysis. *Journal of Financial Research*, 42(3), 449–489.

[41] Hassanein A., Bani-Mustafa A., & Nimer K. (2024). A country’s culture and reporting of sustainability practices in energy industries: does a corporate sustainability committee matter?. *Humanities and Social Sciences Communications*, 11(1), 1–12.

[42] Elyasiani E., & Zhang L. (2015). Bank holding company performance, risk, and “busy” board of directors. *Journal of Banking & Finance*, 60, 239–251.

[43] Kim Y., Lim J., & Qin J. (2022). Board networks and audit quality. *Journal of Corporate Accounting & Finance*, 33(3), 140–148.

[44] Ahn S., Jiraporn P., & Kim Y. S. (2010). Multiple directorships and acquirer returns. *Journal of Banking & Finance*, 34(9), 2011–2026.

[45] Alawaqleh Q. A., Almasria N. A., & ALSAWALHAH J. M. (2021). The effect of board of directors and CEO on audit quality: Evidence from listed manufacturing firms in Jordan. *The Journal of Asian Finance*, *Economics and Business*, 8(2), 243–253.

[46] Bazrafshan A., & Hesarzadeh R. (2022). Board busyness and firm productivity. *Personnel Review*, 51(3), 1138–1168.

[47] Harymawan I., Nasih M., Ratri M. C., & Nowland J. (2019). CEO busyness and firm performance: evidence from Indonesia. *Heliyon*, 5(5)1–9. doi: 10.1016/j.heliyon.2019.e01601 31193196 PMC6522657

[48] Pathan S., Wong P. H., & Benson K. (2019). How do ‘busy’and ‘overlap’directors relate to CEO pay structure and incentives?. *Accounting & Finance*, 59(2), 1341–1382.

[49] Cooper E., & Uzun H. (2012). Directors with a full plate: the impact of busy directors on bank risk. *Managerial Finance*, 38(6), 571–586.

[50] Hamed R., Al-Shattarat W., Mahmood F., Al-Shattarat B., & Hassanein A. (2023). Does corporate sustainable development still promote corporate financial performance during global crises? Empirical Study from China. *Cogent Business & Management*, 10(1), 2190195.

[51] Chandren S., Qaderi S. A., & Ghaleb B. A. A. (2021). The influence of the chairman and CEO effectiveness on operating performance: *Evidence from Malaysia*. *Cogent Business & Management*, 8(1), 1935189.

[52] Ong T., & Djajadikerta H. G. (2020). Corporate governance and sustainability reporting in the Australian resources industry: an empirical analysis. *Social Responsibility Journal*, 16(1), 1–14.

[53] Ruigrok W., Peck S., & Tacheva S. (2007). Nationality and gender diversity on Swiss corporate boards. *Corporate governance*: *an international review*, 15(4), 546–557.

[54] Harris I. C., & Shimizu K. (2004). Too busy to serve? An examination of the influence of overboarded directors. *Journal of Management Studies*, 41(5), 775–798.

[55] Elsayed N., & Hassanein A. (2024). Is voluntary risk disclosure informative? The role of UK firm-level governance. *International Journal of Productivity and Performance Management*, 73(6), 1826–1855.

[56] Boshnak H. A. (2022). Determinants of corporate social and environmental voluntary disclosure in Saudi listed firms. *Journal of Financial Reporting and Accounting*, 20(3/4), 667–692.

[57] Yildirim H. H. (2021). Panel Data Analysis. *Handbook of research on emerging theories*, *models*, *and applications of financial econometrics*, 375–396.

[58] Al-Abbas M. (2009). Corporate governance and earnings management: An empirical study of the Saudi market. *Journal of American Academy of Business*, *Cambridge*, 15(1), 301–310.

[59] McCullagh P. (2019). *Generalized linear models*. Routledge

[60] Florenzcia Y., Juniarti J., & Christiawan Y. J. (2022). Value relevance, sustainability reporting award, and board structure: An influence and analysis of value relevance. *Binus Business Review*, 13(2), 171–181.

[61] Jaffar R., Abu N. A., Hassan M. S., & Rahmat M. M. (2023). Value relevance of board attributes: The mediating role of key audit matter. *International Journal of Financial Studies*, 11(1), 41. doi: 10.3390/ijfs11010041

[62] Badu E., & Assabil E. (2022). Board composition and value relevance of Ghanaian firms: a seemingly unrelated regression approach. *Journal of Economic and Administrative Sciences*, 38(4), 529–543.

[63] Liu S. P., & García Lara J. M. (2023). Do price-earnings multiples for firms with patterns of increasing earnings vary with the quality of the earnings pattern?. *Spanish Journal of Finance and Accounting/Revista Española de Financiación y Contabilidad*, 52(4), 461–498.

[64] Barth M. E., Li K., & McClure C. G. (2023). Evolution in value relevance of accounting information. *The Accounting Review*, 98(1), 1–28.

[65] Jara M., López-Iturriaga F., San-Martín P., & Saona P. (2019). Corporate governance in Latin American firms: Contestability of control and firm value. *BRQ Business Research Quarterly*, 22(4), 257–274.

[66] Kwon S. H., & Wang G. (2020). The change in the value relevance of accounting information after mergers and acquisitions: evidence from the adoption of SFAS 141 (R). *Accounting & Finance*, 60(3), 2717–2757.

[67] Baccouche S., Hadriche M., & Omri A. (2013). The impact of audit committee multiple-directorships on earnings management: Evidence from France. *Journal of Applied Business Research*, 29(5), 1333.

[68] Baccouche S., Hadriche M., & Omri A. (2014). Multiple directorships and board meeting frequency: Evidence from France. *Applied Financial Economics*, 24(14), 983–992.

[69] Chen P., & Hao Y. (2022). Digital transformation and corporate environmental performance: The moderating role of board characteristics. *Corporate Social Responsibility and Environmental Management*, 29(5), 1757–1767.

[70] Pandey R., Vithessonthi C., & Mansi M. (2015). Busy CEOs and the performance of family firms. *Research in International Business and Finance*, 33, 144–166.

[71] Carpenter M. A., & Westphal J. D. (2001). The strategic context of external network ties: Examining the impact of director appointments on board involvement in strategic decision making. *Academy of Management journal*, 44(4), 639–660.

[72] Myers, L., Schmardebeck, R. & Slavov, S. (2021) Audit committee chair succession and financial reporting quality: does firm-specific knowledge matter? Working paper. Available from: https://ssrn.com/abstract=3839962.

[73] Méndez C. F., Pathan S., & García R. A. (2015). Monitoring capabilities of busy and overlap directors: Evidence from Australia. *Pacific-Basin Finance Journal*, 35, 444–469.

[74] Tanyi P. N., & Smith D. B. (2015). Busyness, expertise, and financial reporting quality of audit committee chairs and financial experts. *Auditing*: *A Journal of Practice & Theory*, 34(2), 59–89.

[75] Ahmed K., Hossain M., & Adams M. B. (2006). The effects of board composition and board size on the informativeness of annual accounting earnings. *Corporate governance*: *an international review*, 14(5), 418–431.

[76] Birindelli G., Dell’Atti S., Iannuzzi A. P., & Savioli M. (2018). Composition and activity of the board of directors: Impact on ESG performance in the banking system. *Sustainability*, 10(12), 4699.

[77] Chau G., & Gray S. J. (2010). Family ownership, board independence and voluntary disclosure: Evidence from Hong Kong. *Journal of International Accounting*, *Auditing and Taxation*, 19(2), 93–109.

[78] Fariha R., Hossain M. M., & Ghosh R. (2022). Board characteristics, audit committee attributes and firm performance: empirical evidence from emerging economy. *Asian Journal of Accounting Research*, 7(1), 84–96.

[79] De Andres P., Azofra V., & Lopez F. (2005). Corporate boards in OECD countries: Size, composition, functioning and effectiveness. *Corporate Governance*: *An International Review*, 13(2), 197–210.

[80] Laksmana I. (2008). Corporate board governance and voluntary disclosure of executive compensation practices. *Contemporary accounting research*, 25(4), 1147–1182.

[81] Lipton M., & Lorsch J. W. (1992). A modest proposal for improved corporate governance. *The business lawyer*, 59–77.

[82] Conger J. A., Finegold D., & Lawler E. E. (1998). Appraising boardroom performance. *Harvard business review*, 76, 136–164. 10176916

[83] Bruynseels L., & Cardinaels E. (2014). The audit committee: Management watchdog or personal friend of the CEO?. *The accounting review*, 89(1), 113–145.

[84] Hanlon D., Khedmati M., & Lim E. K. (2019). Boardroom backscratching and audit fees. *Auditing*: *A Journal of Practice & Theory*, 38(2), 179–206.

[85] Cassell C. A., Myers L. A., Schmardebeck R., & Zhou J. (2018). The monitoring effectiveness of co‐opted audit committees. *Contemporary Accounting Research*, 35(4), 1732–1765.

[86] Christensen B. E., Glover S. M., Omer T. C., & Shelley M. K. (2016). Understanding audit quality: Insights from audit professionals and investors. *Contemporary Accounting Research*, 33(4), 1648–1684.

[87] Saidu M., & Aifuwa H. O. (2020). Board characteristics and audit quality: The moderating role of gender diversity. *International Journal of Business & Law Research*, 8(1), 144–155.

[88] Azizkhani M., Hossain S., & Nguyen M. (2023). Effects of audit committee chair characteristics on auditor choice, audit fee and audit quality. *Accounting & Finance*, 63(3), 3675–3707.

[89] Zaman M., Hudaib M., & Haniffa R. (2011). Corporate governance quality, audit fees and non‐audit services fees. *Journal of Business Finance & Accounting*, 38(1‐2), 165–197.

[90] Beasley M. S., Carcello J. V., Hermanson D. R., & Neal T. L. (2009). The audit committee oversight process. *Contemporary Accounting Research*, 26(1), 65–122.

[91] Free C., Trotman A. J., & Trotman K. T. (2021). How audit committee chairs address information-processing barriers. *The Accounting Review*, 96(1), 147–169.

[92] Dhaliwal D. A. N., Naiker V. I. C., & Navissi F. (2010). The association between accruals quality and the characteristics of accounting experts and mix of expertise on audit committees. *Contemporary accounting research*, 27(3), 787–827.

[93] DeFond M. L., Hann R. N., & Hu X. (2005). Does the market value financial expertise on audit committees of boards of directors?. *Journal of accounting research*, 43(2), 153–193.

[94] Cohen J. R., Hoitash U., Krishnamoorthy G., & Wright A. M. (2014). The effect of audit committee industry expertise on monitoring the financial reporting process. *The Accounting Review*, 89(1), 243–273.

[95] Ammer M. A., Aliedan M. M., & Alyahya M. A. (2020). Do corporate environmental sustainability practices influence firm value? The role of independent directors: Evidence from Saudi Arabia. *Sustainability*, 12(22), 9768.

[96] Rubino F., & Napoli F. (2020). What impact does corporate governance have on corporate environmental performances? An empirical study of Italian listed firms. *Sustainability*, 12(14), 5742.

[97] Chebbi K., & Ammer M. A. (2022). Board composition and ESG disclosure in Saudi Arabia: The moderating role of corporate governance reforms. *Sustainability*, 14(19), 12173.

[98] Al-Duais S. D., Qasem A., Wan-Hussin W. N., Bamahros H. M., Thomran M., & Alquhaif A. (2021). CEO characteristics, family ownership and corporate social responsibility reporting: The case of Saudi Arabia. *Sustainability*, 13(21), 12237.

[99] Almulhim A. A., & Aljughaiman A. A. (2023). Corporate sustainability and financial performance: The moderating effect of CEO characteristics. *Sustainability*, 15(16), 12664.

[100] Singh A., Singh P., & Shome S. (2022). ESG–CFP linkages: A review of its antecedents and scope for future research. *Indian Journal of Corporate Governance*, 15(1), 48–69.

[101] Hayn C. (1995). The information content of losses. *Journal of Accounting and Economics*, 20(2), 125–153. doi: 10.1016/0165-4101(95)00397-2

[102] Roychowdhury S., Shroff N., & Verdi R. S. (2019). The effects of financial reporting and disclosure on corporate investment: A review. *Journal of Accounting and Economics*, 68(2–3), 101246.

[103] Francis J. R. (2004). What do we know about audit quality?. *The British accounting review*, 36(4), 345–368.

[104] Francis J., & Schipper K. (1999). Have financial statements lost their relevance?. Journal of Accounting Research, 37(2), 319–352. doi: 10.2307/2491412

[105] Adams R. B., & Ferreira D. (2007). A theory of friendly boards. *The journal of finance*, 62(1), 217–250.

[106] Alzoubi E. S. S. (2014). Board characteristics and financial reporting quality: Evidence from Jordan. *Corporate Ownership and Control*, 11(3), 8–29.

[107] Imam M. O., & Jaber M. I. (2014). Earning management of IPOs in Bangladesh-test of value relevance hypotheses: Evidence from Dhaka Stock Exchange. *Independent Business Review*, 7(2), 1–31.

[108] Imhanzenobe J. (2022). Value relevance and changes in accounting standards: A review of the IFRS adoption literature. *Cogent Business & Management*, 9(1), 2039057.

[109] Lisic L. L., Myers L. A., Seidel T. A., & Zhou J. (2019). Does audit committee accounting expertise help to promote audit quality? Evidence from auditor reporting of internal control weaknesses. *Contemporary Accounting Research*, 36(4), 2521–2553. doi: 10.1111/1911-3846.12517

[110] Mohammed I. A., Che-Ahmad A., & Malek M. (2019). Regulatory changes, board monitoring and earnings management in Nigerian financial institutions. *DLSU Business & Economics Review*, 28(2), 152–168.

[111] Soliman M. M., & Ragab A. A. (2014). Audit committee effectiveness, audit quality and earnings management: an empirical study of the listed firms in Egypt. *Research Journal of Finance and Accounting*, 5(2), 155–166.

[112] Abbadi S. S., Hijazi Q. F., & Al-Rahahleh A. S. (2016). Corporate governance quality and earnings management: Evidence from Jordan. *Australasian Accounting*, *Business and Finance Journal*, 10(2), 54–75.

[113] Sun J., Lan G., & Liu G. (2014). Independent audit committee characteristics and real earnings management. Managerial Auditing Journal, 29(2), 153–172.

[114] Collins D. W., Maydew E. L., & Weiss I. S. (1997). Changes in the value-relevance of earnings and book values over the past forty years. *Journal of Accounting and Economics*, 24(1), 39–67.

[115] Acaranupong K. (2021). International Financial Reporting Standards Convergence and Value Relevance of Accounting Information: Evidence from ASEAN. *Asian Journal of Business and Accounting*, 14(2), 31–68.

[116] Dimitropoulos P., & Koronios K. (2021). Corporate Environmental Responsibility and Earnings Value Relevance. Corporate Environmental Responsibility, *Accounting and Corporate Finance in the EU*: *A Quantitative Analysis Approach*, 197–213.

[117] Chehade S., & Procházka D. (2024). Value relevance of accounting information in an emerging market: the case of IFRS adoption by non-financial listed firms in Saudi Arabia. *Journal of Accounting in Emerging Economies*, 14 *(*2*)*, 220–246. doi: 10.1108/JAEE-06-2022-0165

[118] DeFond M., & Zhang J. (2014). A review of archival auditing research. *Journal of accounting and economics*, 58(2–3), 275–326.

[119] Krishnan G.V. & Visvanathan G. (2008) Does the SOX definition of an accounting expert matter? The associ- ation between audit committee directors’ accounting expertise and accounting conservatism. *Contemporary Accounting Research*, 25(3), 827–858.

[120] Abernathy J. L., Beyer B., Masli A., & Stefaniak C. (2014). The association between characteristics of audit committee accounting experts, audit committee chairs, and financial reporting timeliness. *Advances in Accounting*, 30(2), 283–297.

